# Domain Wall Patterning and Giant Response Functions in Ferrimagnetic Spinels

**DOI:** 10.1002/advs.202101402

**Published:** 2021-11-01

**Authors:** Lazar L. Kish, Alex Thaler, Minseong Lee, Alexander V. Zakrzewski, Dalmau Reig‐i‐Plessis, Brian A. Wolin, Xu Wang, Kenneth C. Littrell, Raffi Budakian, Haidong Zhou, Zheng Gai, Matthias D. Frontzek, Vivien S. Zapf, Adam A. Aczel, Lisa DeBeer‐Schmitt, Gregory J. MacDougall

**Affiliations:** ^1^ Department of Physics and Materials Research Laboratory University of Illinois at Urbana‐Champaign Urbana IL 61801 USA; ^2^ National High Magnetic Field Laboratory Los Alamos National Laboratory Los Alamos NM 87544 USA; ^3^ Department of Physics and Astronomy and Quantum Matter Institute University of British Columbia Vancouver British Columbia V6T 1Z1 Canada; ^4^ Department of Physics and Astronomy University of Tennessee Knoxville Tennessee 37996 USA; ^5^ Department of Physics and Astronomy University of Waterloo Waterloo Ontario N2L 3G1 Canada; ^6^ Center for Nanophase Materials Sciences Oak Ridge National Laboratory Oak Ridge TN 37831 USA; ^7^ Neutron Scattering Division Oak Ridge National Laboratory Oak Ridge TN 37831 USA

**Keywords:** domain walls, magnetodielectrics, magnetoelastics, magnetostructural transitions, small‐angle neutron scattering

## Abstract

The manipulation of mesoscale domain wall phenomena has emerged as a powerful strategy for designing ferroelectric responses in functional devices, but its full potential is not yet realized in the field of magnetism. This work shows a direct connection between magnetic response functions in mechanically strained samples of Mn_3_O_4_ and MnV_2_O_4_ and stripe‐like patternings of the bulk magnetization which appear below known magnetostructural transitions. Building off previous magnetic force microscopy data, a small‐angle neutron scattering is used to show that these patterns represent distinctive magnetic phenomena which extend throughout the bulk of two separate materials, and further are controllable via applied magnetic field and mechanical stress. These results are unambiguously connected to the anomalously large magnetoelastic and magnetodielectric response functions reported for these materials, by performing susceptibility measurements on the same crystals and directly correlating local and macroscopic data.

## Graphical abstract

Magnetostructural transitions in the ferrimagnetic spinels Mn_3_O_4_ and MnV_2_O_4_ are linked to increased spin–lattice coupling, from which novel magnetic domain structures emerge on the mesoscale. Small‐angle neutron scattering measures the response of these domain patterns to applied magnetic field and stress. Correlations between scattering and bulk probes demonstrate tuning of macroscopic response functions via alterations to the domain structure.

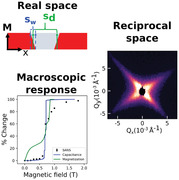

## 1 Introduction

Domain walls,^[^ ^1^, ^2^, ^3^ ^]^ dislocations,^[^ ^4^ ^]^ and other moving defects^[^ ^5^ ^]^ are critically important to the response functions of applied materials, and consequently, control of such defects has been exploited to develop materials with tuneable properties for use in flexible, next‐generation devices.^[^ ^5^ ^]^ One notable example is the nanometer‐lengthscale domain wall patterning which is observed to form in some ferroelastic materials as a means to reduce the energy associated with long‐range strain fields.^[^ ^2^, ^6^, ^7^ ^]^ Similar stripe‐like domains often form in ferroelectric materials by grace of strain and strong electromechanical couplings.^[^ ^2^, ^6^, ^7^ ^]^ In ferroelectrics, these domain walls can be charged and conducting,^[^ ^8^ ^]^ which can drastically alter the macroscopic conductive and capacitive response functions.^[^ ^9^ ^]^ As such, the control of domain patterning in these materials has been extensively examined in pursuit of new optoelectronic and capacitive memory devices.^[^ ^10^ ^]^ Even in the absence of a net ferroelectric moment, heterogeneous local permittivity near domain walls or external interfaces can lead to useful dielectric response functions by the Maxwell–Wagner effect.^[^ ^11^, ^12^ ^]^

The full implications of co‐existing magnetic degrees of freedom for mesoscale phenomena are far from understood, but recent experiments hint at exciting new possibilities.^[^ ^13^ ^]^ Single phase^[^ ^14^ ^]^ and composite^[^ ^15^ ^]^ multiferroics have concomitant electric and magnetic moments and have long elicited great interest from spintronics and applied physics communities. Much like pure ferroelectrics, investigation of multiferroic materials has often revealed the existence of mesoscale domain patterning^[^ ^3^ ^]^ and associated colossal magnetodielectric response functions,^[^ ^14^ ^]^ leading to suggestions of field‐tunable capacitive elements or cavity resonators.^[^ ^15^, ^16^ ^]^ An enhanced magnetodielectric response is often associated with magnetic transitions^[^ ^12^ ^]^ and frequently considered a signature of coupled ferroelectric and ferromagnetic orders.^[^ ^14^ ^]^ Much like above, however, enhanced magnetodielectric couplings important to many potential applications can arise even in the absence of ferroelectricity, through local spin‐phonon coupling or extrinsic mechanisms.^[^ ^12^, ^17^ ^]^

Several potential examples can be found in the literature on ferrimagnetic spinel oxides. These materials are notable for strong spin–lattice coupling and the prevalence of low‐temperature magnetostructural transitions.^[^ ^18^ ^]^ Often these low‐temperature states are characterized by giant magnetoelastic and magnetodielectric response functions,^[^ ^19^, ^20^, ^21^, ^22^, ^23^, ^24^ ^]^ though reports are inconsistent and there is very little agreement as to the origin of these behaviors. Recent studies by members of our collaboration using magnetic force microscopy (MFM)^[^ ^25^ ^]^ and others using transmission electron microscopy (TEM)^[^ ^26^, ^27^ ^]^ have revealed the existence of stripe‐like magnetization domains in the same temperature region in some spinels when subjected to applied stress. Initial estimates indicate proper consideration of these stripe‐like domains is essential to account for the magnitude of the bulk magnetization data in the same crystals,^[^ ^25^ ^]^ though it is difficult to make quantitative predictions based on surface investigations alone. To our knowledge, there has been no systematic study correlating the behavior of mesoscale domains with the reported anomalous response functions in these materials.

In the current paper, we present a joint magnetization, magnetocapacitance, and small‐angle neutron scattering (SANS) study which connects anomalous macroscopic properties to the field response of stripe‐like magnetic domain wall patterning in two specific spinel ferrimagnets: Mn_3_O_4_ (MMO) and MnV_2_O_4_ (MVO). MMO is the prototypical tetragonal spinel, with a Jahn–Teller transition at 1440 K,^[^ ^28^ ^]^ and three magnetic transitions^[^ ^29^, ^30^ ^]^ at 42 K ($T_{\text{FM}}^{\text{MMO}}$), 39 K ($T_{\text{IC}}^{\text{MMO}}$), and 33 K ($T_{\text{MS}}^{\text{MMO}}$). The 33 K transition was recently shown to have a structural component,^[^ ^31^ ^]^ and marks the onset of stripe‐like magnetic domains at low temperature.^[^ ^25^ ^]^ MVO has a magnetic‐ordering transition at 56 K ($T_{\text{FM}}^{\text{MVO}}$) and an orbital‐ordering transition at 53 K ($T_{\text{MS}}^{\text{MVO}}$), which increases coupling between spins and the lattice.^[^ ^23^, ^32^ ^]^ At the lowest temperatures, MMO features a net ferrimagnetic moment along its local orthorhombic (*L*_Orth._) <110 > directions and a hard axis in the global [001] direction,^[^ ^33^ ^]^ while MVO in contrast has moments along its local tetragonal (*L*_Tet._) <001 > directions. (We will refer to all crystallographic directions using the high‐symmetry cubic setting, with preceding labels *L*_Tet._ and *L*_Orth._ when referring to local crystal axes of individual domains, and the qualifier ”global” when referring to macroscopic coordinates. Section S1, Supporting Information further details our crystallographic conventions.) In both materials, the lowest‐temperature phases have been associated with large magnetoelastic and magnetodielectric response functions^[^ ^20^, ^21^, ^24^, ^34^, ^35^ ^]^ and stripe‐like magnetic domains in our previous MFM study.^[^ ^25^ ^]^

Our current SANS data expand our understanding of these mesoscale patterns in several important ways. First, as a bulk probe of matter, neutron scattering was able to confirm that the low‐temperature stripe‐like domains were not simply surface phenomena, but extended to the bulk of each material. As a reciprocal space probe, SANS was able to comment on the orientation and average distribution of wall separations throughout the sample, a feat not possible with MFM due to the limited real‐space scan range. The relative speed of SANS measurements allowed us to rapidly perform parametric studies to correlate the evolution of magnetization patterns in reciprocal space with specific features in time‐dependent magnetization and magnetodielectric data from the same crystals. These results, coupled with the known scattering cross‐section of SANS, opens up new research possibilities wherein unique domain wall patterns can be tuned and characterized with neutron scattering to create predictable, user‐defined functional behaviors in a wide range of correlated materials.

## 2 Results

### 2.1 Characterization of Domain Structure

**Figure 1:**
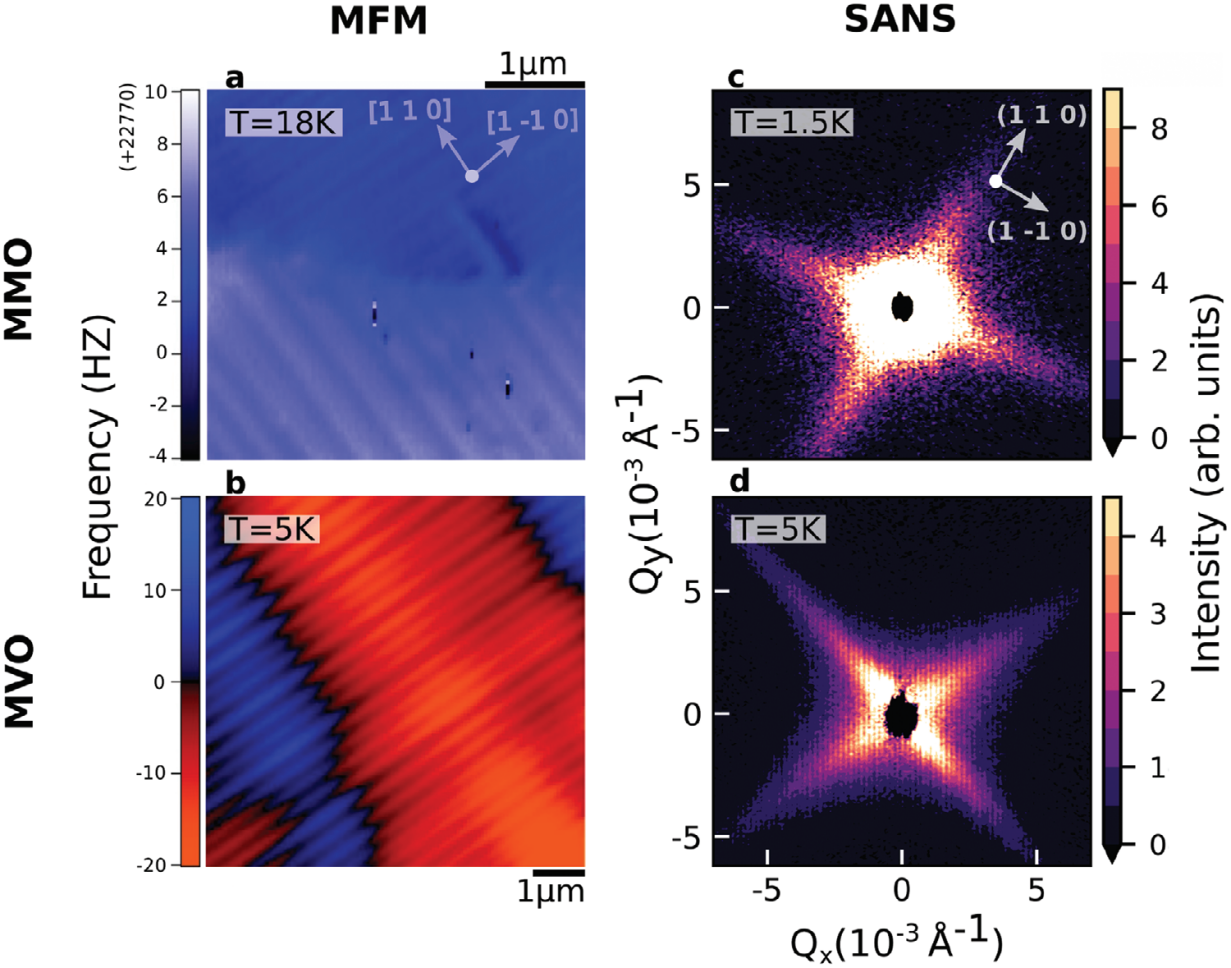
Magnetic domain structure via MFM and SANS. MFM images of stripe patterning on the (00L) surfaces of a) MMO and b) MVO, taken below respective structural transitions. Arrows show approximate crystallographic orientations with respect to the stripes. Reported MVO frequency is shift with respect to natural resonance frequency of the probe. SANS patterns from the (HK0) scattering plane of c) MMO at *T* = 1.5 K and d) MVO at *T* = 5 K, with the measured scattering in the paramagnetic phase subtracted off as background.

The overarching goal of the current study is to establish a causal connection between reports of anomalous magnetic response behaviors in ferrimagnetic spinel materials and recent observations by members of our collaboration^[^ ^25^ ^]^ with MFM of an unusual stripe‐like patterning of local magnetization. An essential first step was to establish that the surface phenomena observed with MFM extended throughout the bulk of the relevant materials, to characterize average behaviors and to acquire sufficient parametric data which would allow for comparison to bulk measurements. As described above, our experimental technique of choice for this purpose was SANS, which can be used to detect variations in local magnetization with typical lengthscale 0.5–200 nm. Measurements were performed using single‐crystal samples from the same batch as studied in Wolin et al.,^[^ ^25^ ^]^ and a comparison of data from the two techniques can be found in **Figure** **1**.

In Figure 1a,b, we show real‐space MFM images of zero‐field cooled magnetic domain patterns below the respective magnetostructural transitions in MMO (MVO) single crystals, previously published in Wolin et al.^[^ ^25^ ^]^ MFM is a cantilever‐based scanning probe which measures the out‐of‐plane surface magnetic field at the position of a probe tip. The images shown can be thought of as maps of the out‐of‐plane component of the local magnetization of the sample, measured with 50 nm spatial resolution. On the surface of each material, MFM reveals 100 nm‐lengthscale modulations of the underlying ferrimagnetism, though the wall separation is seen to vary even over the 3 *μm* mapped region of the crystal. The propagation of the walls are aligned parallel to the crystallographic (global) <110 > directions. The morphology and oriented nature of these domains differ from expectations for domains in a typical ferromagnet and imply a coupling to the crystal structure akin to ferroelastic domains.^[^ ^2^ ^]^ This idea is discussed in more detail in subsequent sections.

By identifying reciprocal space signatures of the stripe domains, we will show in this section that they are not only surface effects but persist through the bulk of our single‐crystal samples. Figure 1c,d displays directly comparable SANS intensities in MMO (MVO) at 1.5 K (5 K) and zero field, after subtraction of patterns from the high‐temperature paramagnetic phase. The major features seen here can be mapped directly to the hierarchical magnetic domain structure observed in the real‐space MFM maps. The stripe patterns manifest distinctly as anisotropic fins of intensity stretching along global {110} directions, the same directionality as the MFM domain walls and, as we will show in this section, reflecting the same characteristic lengthscales.

It is interesting to note that the novel fin scattering in the two materials points in the same crystallographic direction. This implies that the stripe walls in the two materials are oriented similarly, despite the difference in both ordered moment direction and low‐temperature crystal structure. This can be understood if the measured magnetization domains are reflective of an underlying crystallographic twinning structure. By analogy with transformation twin walls in other materials,^[^ ^2^ ^]^ one would expect the chief determinant of the wall orientation to be strain induced in the sample during the (ferroelastic) structural transitions. Such twin walls often orient themselves normal or parallel to a lost mirror plane in the low‐symmetry phase. In MVO, where the distortions at the magnetostructural transition occur along *L*_Tet._ {001} directions, our observations are consistent with, for example, *L*_Tet._ (1 0 0) and *L*_Tet._ (0 1 0) c‐axis domains forming boundaries parallel to the global {110} set of planes. A similar picture applies for MMO,^[^ ^36^ ^]^ where the low‐field orthorhombic phase has its in‐plane short axis along the *L*_Orth._ {100} direction (a global c‐axis direction having been determined during growth).

Since both materials possess a fourfold symmetry about the c‐axis above their ordering transitions, domain walls are expected to develop in the full set of {110} directions as we cool below the transitions. This would naturally lead to the observed cross‐shape as the superposition of fin scattering from quasi‐1D stripe patterning in both [1 1 0] and [1 ‐1 0] directions. In contrast, alternative 2D geometries which might also produce similar scattering, also predict scattering features which are inconsistent with our data. For example, cuboid domains^[^ ^37^ ^]^ are expected to display significant scattering in the diagonal directions such as {010}, in addition to scattering along {110} directions due to comparable lengthscales of domain size. Such scattering was not readily observed in this low‐Q region, implying that the domains retain their highly anisotropic stripe shape in the bulk.

**Figure 2:**
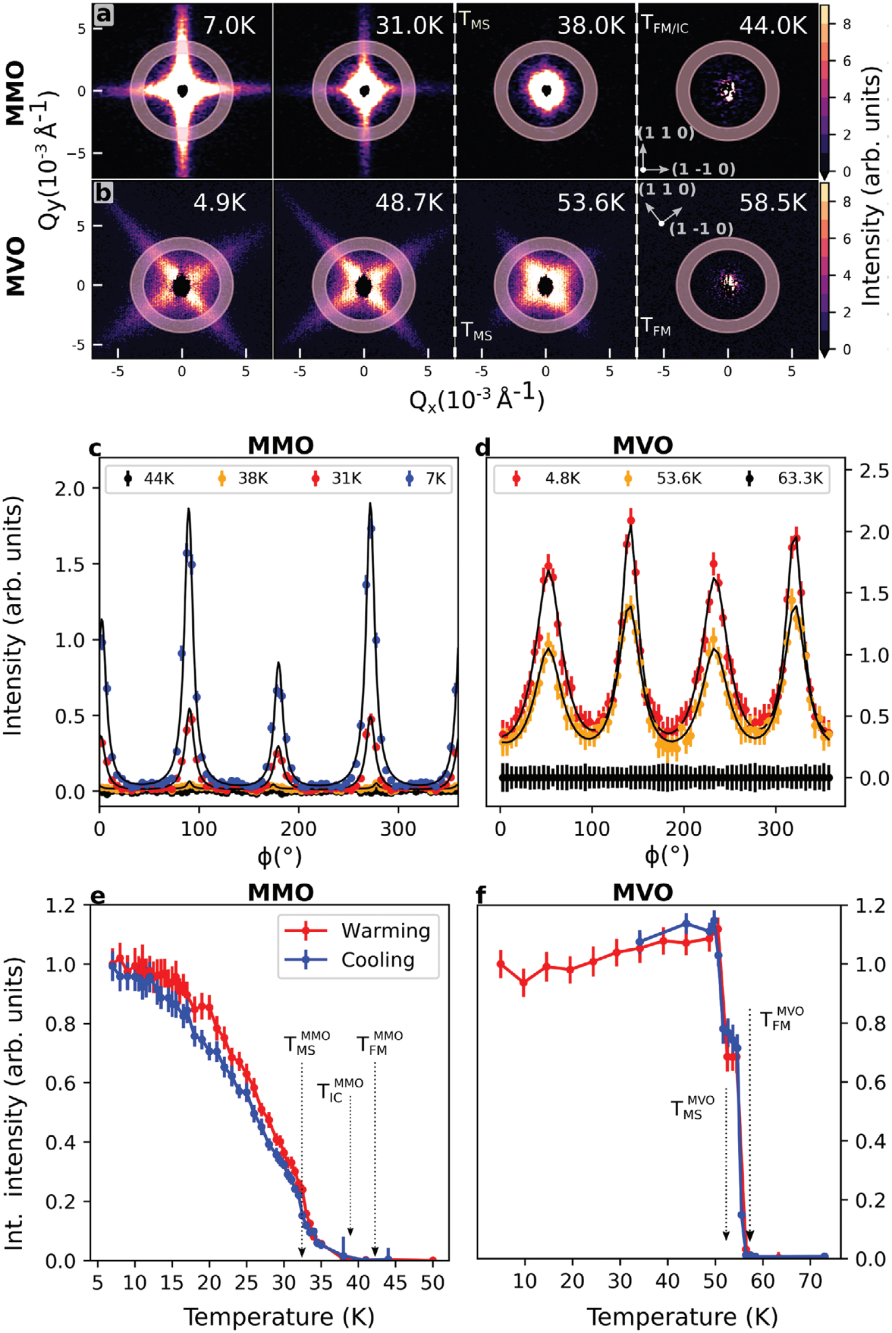
Temperature dependence of SANS intensity. Raw scattering intensity from SANS in the (HK0) plane at selected temperatures above and below the phase transitions in a) MMO and () MVO down to lowest attained temperatures of 7 and 4.9 K, respectively. Transitions are marked and labeled by dashed lines between the panels. Annular Q‐cuts in c) MMO and d) MVO, with an integration range of 0.003–0.004 Å^−1^ with the associated Lorentzian fits superimposed on the data. Integrated intensity of the fin scattering plotted for e) MMO and f) MVO as a function of temperature on both warming and cooling, with marked transitions. Error bars represent one standard deviation in panels (c, d) and one standard error as determined from nonlinear least‐squares fitting in panels (e, f).

The interpretation of these scattering features are further reinforced by tracking the temperature dependence. **Figure** **2**a,b shows the progression of the 2D SANS patterns in each material, as temperature is lowered below their respective ferrimagnetic ordering (*T*_FM_) and magnetostructural (*T*_MS_) transitions. Both show weak scattering near *Q* = 0 at highest temperatures, consistent with a paramagnetic state.

In MMO, the scattering also contains an isotropic component. This is associated with the 1–10 micron spin‐only domains that typically form in ferromagnetic materials to minimize demagnetization energy.^[^ ^1^, ^38^ ^]^ We will call these “demagnetization domains” to distinguish them from the magnetostructural stripe domains. This isotropic scattering appears immediately below $T_{\text{FM}}^{\text{MMO}}$ (as can be seen at 38 K in Figure 2a), and the anisotropic features develop as a redistribution of this intensity below $T_{\text{MS}}^{\text{MMO}}$.

In contrast, the MVO scattering patterns show no temperature region with only isotropic scattering. If present, the features are less prevalent in SANS patterns for MVO. This is potentially due to the larger size of the demagnetization domains as shown by MFM.^[^ ^25^ ^]^ Instead, the anisotropic scattering appears immediately at $T_{\text{FM}}^{\text{MVO}}$. The fin scattering then jumps in intensity upon cooling through $T_{\text{MS}}^{\text{MVO}}$, with a shift in scattering away from the origin. MFM reports that this region is characterized by sporadic stripe development at zero field,^[^ ^25^ ^]^ consistent with a cubic–tetragonal phase mixture, as has been reported elsewhere.^[^ ^39^ ^]^

To quantify these trends and facilitate detailed modeling, annular cuts were taken for *Q* from 0.003–0.004 Å^−1^ (the shaded regions in Figure 2a,b) and fit to Lorentzian transverse profiles, as shown in Figure 2c,d. The peaks in this figure are positioned exactly 90 degrees apart, as expected due to the fourfold symmetry of the fin scattering. The slightly lower intensity in two of the (horizontal) fins is because the crystal alignment was optimized to maximize scattering in the other (vertical) fins. Subsequent analysis focuses solely on the vertical peaks, and so this apparent deviation from fourfold symmetry does not affect the conclusions of the paper.

The integrated intensities of the Lorentzian fits are plotted in Figure 2e,f on both warming and cooling. The results show that the fins appear in MMO at $T_{\text{MS}}^{\text{MMO}}$ and saturate below 10 K. The intermediate spin spiral phase^[^ ^29^, ^30^ ^]^ between $T_{\text{MS}}^{\text{MMO}}$ and $T_{\text{IC}}^{\text{MMO}}$ also sees a small Curie‐like tail of fin intensity that dies off when warming above the latter transition. In MVO, the anisotropy appears in two sharp steps at $T_{\text{FM}}^{\text{MVO}}$ and $T_{\text{MS}}^{\text{MVO}}$. These trends and the weak temperature hysteresis are largely in line with a collection of Raman spectroscopy, diffraction, and macrostrain measurements in the literature tracking the magnetostructural transitions in each material.^[^ ^23^, ^24^, ^32^, ^35^, ^40^ ^]^

**Figure 3:**
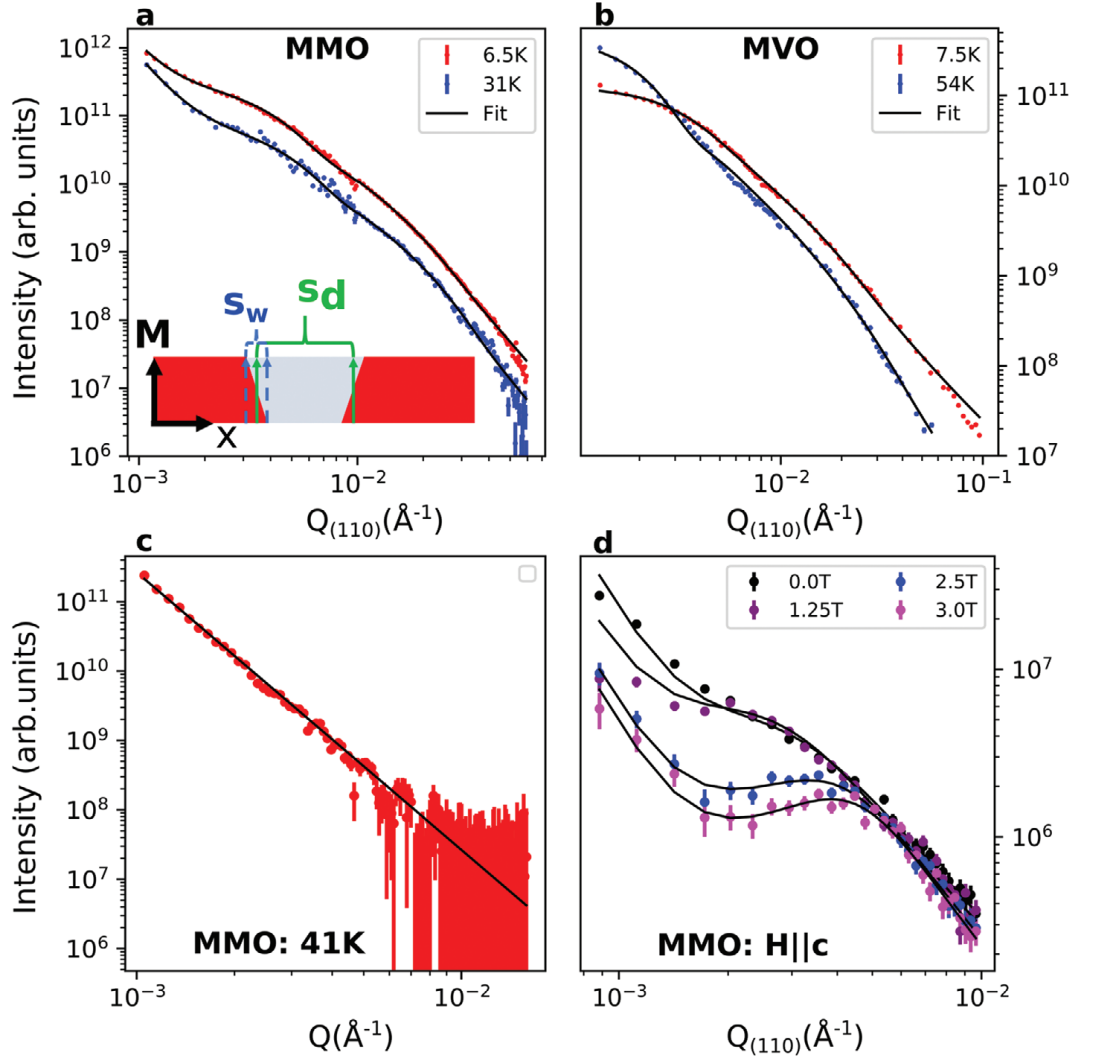
SANS intensity momentum dependence. Dependence of SANS intensity on *Q*_(110)_ for a) MMO and b) MVO with fits to the 1D stripe model described in the text superimposed on the data. The inset of (a) shows a real‐space schematic of the model, with stripe domain size and wall size *s*_*d*_ and *s*_*w*_. c Isotropic average of intensity versus *Q* in MMO at 41 K, showing Porod scattering arising from demagnetization domain walls. d) Field‐induced redistribution of MMO low‐*Q* intensity versus *Q*_(110)_. Error bars represent one standard deviation.

It is important to note that intensity variations in the annular profiles can be affected both by changes in the number of domain walls, which alters the overall intensity, as well as changes in the average domain size, which will alter the radial profile of the intensity distribution. **Figure** **3** displays the dependence of the scattering intensity on momentum *Q* in various directions for both materials. Figure 3a,b shows intensity profiles of rectangular line cuts along global (110) at temperatures where fin scattering is visible, with the transverse width varying from 0.002 to 0.016 Å^−1^ to capture the full breadth of the features using the different instrument settings (Section S2, Supporting Information). These profiles are notable for their subtle curvature on a log–log scale, which can be captured by a simple model equation:

(1) $$\begin{array}{ccc} I(Q_{\left(110\right)}) & = & a\left(\sin c^{2}\left(\frac{sQ_{\left(110\right)}}{2}\right)*G\left(s,s_{d},\sigma_{d}\right)\right)\\ & & \left(\sin c^{2}\left(\frac{sQ_{\left(110\right)}}{2}\right)*G\left(s,s_{w},\sigma_{w}\right)\right). \end{array}$$

This model supposes that individual stripe domains are 1D regions of constant magnetization, with linear domain walls. The domains and their walls have variable widths of *s*_d_ and *s*_w_, respectively, and *a* is a scaling parameter. The 1D domain form factors are convoluted with normalized Gaussian size distributions

(2) $$G=\frac{\exp(-\frac{{\left(s-s_{*}\right)}^{2}}{2\sigma^{2}})}{\sqrt{2\pi }\sigma }$$

with center s_*_ = *s*_d_ (*s*_w_) and width *σ* = *σ*_d_ (*σ*_w_) to account for polydispersity. Additional details are listed in Section S4, Supporting Information . Calculated intensities from this model are included in Figure 3a,b as solid lines, and are seen to describe the scattering data across six orders of magnitude of intensity.

For MMO, we also include a isotropic Porod scattering component (*I* = *b* *Q* ^−4^).^[^ ^38^ ^]^ This component appears in the low‐*Q* region of our data and is typical of ferromagnetic demagnetization domains^[^ ^38^ ^]^ with smooth and isotropically distributed walls. This power law behavior accounts for all of the intensity in the ferrimagnetic temperature region between $T_{\text{FM}}^{\text{MMO}}$ and $T_{\text{MS}}^{\text{MMO}}$, as demonstrated by the isotropically averaged 41 K dataset displayed in Figure 3c.

Fits to this model at the lowest measured temperatures provide a mean stripe width of 86.7 ± 1.7 nm for MMO and 90.5 ± 1.3 nm for MVO, which are broadly consistent with the local MFM observations.^[^ ^25^ ^]^ The domain walls were found to have a width 15.8 ± 0.2 nm for MMO and 15.8 ± 0.5 nm for MVO, below the resolution limit of MFM. Near the magnetostructural transition in ($T_{\text{MS}}^{\text{MMO}}$), fits give a mean stripe width of 72.2 ± 1.7 nm, or slightly smaller than the low‐temperature value. In contrast, the region between the two transitions of MVO ($T_{\text{MS}}^{\text{MVO}}$ and $T_{\text{FM}}^{\text{MVO}}$) shows a motion of fin scattering closer to *Q* = 0 and fits correspondingly yield a larger mean stripe width of 169.0 ± 3.0 nm with a 13.3 ± 1.4 nm wall width. Standard deviations of the size distributions converged to quite large values ($\sigma_{d}\approx 30\%$ of the mean size), explaining in part the lack of strong intensity peaks in our data. Though the intensity distribution in MMO was well fit, our estimate of the domain wall width should be thought of as an upper bound due to potential complications in fully accounting for grain misalignments in this material.

These fits yield size information along the short dimension of the stripe domains (the stripe width). The change to a larger mean stripe width for MVO between its two transitions, $T_{\text{MS}}^{\text{MVO}}$ and $T_{\text{FM}}^{\text{MVO}}$, is accompanied by a noticeable broadening in the direction perpendicular to our rectangular cuts as well. This is visible both in the 2D data (Figure 2b) and annular cuts (Figure 2d), and implies that while domain walls move further apart, the stripe domains also become less well defined along their length.

Together, this tells us that the domain structure is changing across both dimensions in MVO as we vary temperature in the intermediate region. As mentioned in Section 2.1, the orientation of the stripe walls can be understood if the magnetic stripe domains are reflective of an underlying twin structure in the lattice. The energetics of transformation twins are strongly coupled to internal strain fields which develop at structural transitions.^[^ ^41^ ^]^ Thus, the observed dynamics are therefore likely related to a rapid development of spontaneous strain in the system which only stabilizes below $T_{\text{MS}}^{\text{MVO}}$, or to the possibility of a cubic–tetragonal phase mixture.^[^ ^23^, ^39^ ^]^ In MMO, the stripe width shows only a weak temperature dependence, despite the rapid increase in scattering amplitude below $T_{\text{MS}}^{\text{MMO}}$. This seems to imply that domain growth is happening along a direction we cannot observe. Because in this material the fin scattering is sharper and resolution limited along the perpendicular direction, our results are consistent with the domains nucleating and immediately growing to a size surpassing the resolution limitations of the measurement along this perpendicular direction. This is also consistent with our MFM data, which shows stripes nucleating along the perpendicular dimension.^[^ ^25^ ^]^

### 2.2 Response to Applied Field

The strong variation of the local ferrimagnetic order throughout the bulk of the samples inferred from SANS has significant implications for the macroscopic properties of the materials below *T* = *T*_MS_. This strongly motivates a detailed exploration of the effect of applied field, as any strong field response would have immediate consequences for the inferred bulk magnetic susceptibilities in both spin and charge channels.

**Figure 4:**
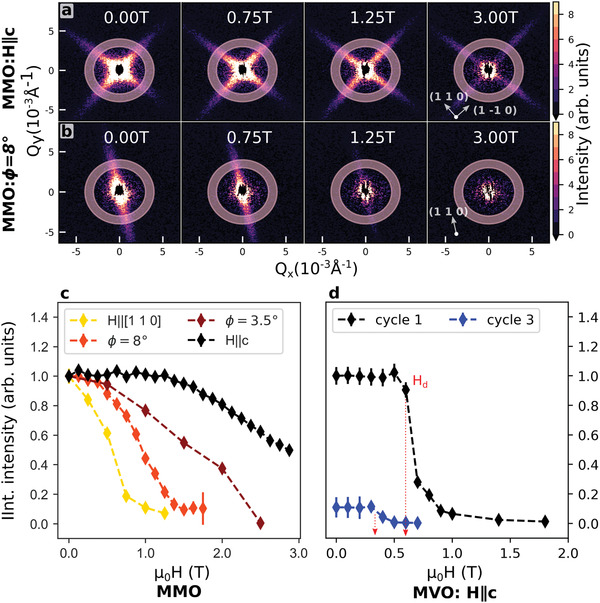
Domain response to applied magnetic field in SANS. Raw 2D SANS data from MMO as a function of increasing field a) parallel to the hard global c axis and b) *ϕ* = 8° misaligned from global c, demonstrating anisotropic response in the fin intensity. c) MMO integrated fin intensity in the range 0.003–0.004 Å^−1^ as a function of field applied along different directions with respect to global c. Intensities were rescaled to match the first data point between orientations. d) MVO integrated fin intensity in the range 0.01–0.12 Å^−1^ as a function of field along global c‐axis normal to sample plate, for two field cycles as described in the text, with equal scaling for the intensities. Error bars in panels (c, d) represent one standard error as determined from nonlinear least‐squares fitting.

In fact, the responsivity of the stripe‐like domains to applied magnetic fields is substantial, as can be seen in **Figures** **3**d and  4. Figure 3d shows the evolution of *I* versus *Q*_(110)_ in MMO at low temperature as magnetic fields are applied in the out‐of‐plane (global) c‐axis direction, along with associated fits. In contrast to relatively featureless low‐field curves, applied fields of a few Tesla are observed to redistribute the scattering intensity until peaks representing distinct spatial correlations are visible. This corresponds to the system evolving from a state with weak correlations to one where stripes are more regularly arranged. The position of the peak at *μ*_0_ *H* = 3 T corresponds to inter‐stripe spacings of ≈300 nm, comparable to distances observed with MFM.^[^ ^25^ ^]^

Representative 2D scattering patterns for MMO at *T* = 2 K are shown in Figure 4a,b for measurements performed both with applied fields H∥c and with the sample tilted at an angle *ϕ* = 8° off‐axis. For the former, the most obvious effect of field is the above‐described sharpening of the fin features, with only a moderate decrease in integrated intensity at the highest field available (3 T). However, if field direction is varied even a few degrees, fin intensity decreases much more rapidly and disappears almost entirely by 1 T. This strong anisotropy presents more clearly in Figure 4c, where we show integrated scattering intensity in the fins versus *H* for four different field orientations. Subsequent removal of applied fields shows no return of fin scattering. These observations are consistent with a picture wherein stripe domain walls begin to move above a “depinning field,” *H*_d_, allowing the system to arrange itself into a homogeneous phase with lower overall energy. In MMO, *H*_d_ is highly anisotropic in terms of field direction with respect to the c‐axis. This effect is similar to the depinning fields which have been reported for structural domains in several ferroelectric materials.^[^ ^42^, ^43^ ^]^ This interpretation is consistent with MFM studies on MMO^[^ ^25^, ^44^ ^]^ and our own magnetization data presented below.

Figure 4d demonstrates a similar field‐induced collapse of stripe domain patterning in MVO. Here, integrated scattering intensity in the fits is plotted as a function of applied field, *H* applied along the global c‐axis. A well‐defined initial plateau yields to a steep drop of integrated fin intensity at H = *H*_d_ with increasing field, much like above. Corresponding 2D patterns are displayed in Section S7, Supporting Information. As an interesting supplemental behavior not seen in MMO, field and temperature cycling of the sample appears to lower both *H*_d_ and the integrated fin intensity in MVO. This manifests as a difference in scattering intensity between cycles 1 and 3 in Figure 4d (spacing in field for cycle 2 was insufficient for a useful comparison). In one “cycle” here, the sample is zero‐field cooled to low temperature, whereupon field is ramped to a high value (>1.2T) during measurement. Field is subsequently removed, and the sample heated above its magnetic‐ordering temperature (>60K) before cooling for further scans. Despite thermally cycling above the transition, the low‐temperature magnetic properties apparently reflects some memory about the number of the thermal history. The combined analysis in Section 2.4 draws a direct connection between these effects and sample strain, which arises via different routes in the two materials.

### 2.3 Comparison to Bulk Response Functions

The detailed temperature and field parameterization obtained above using SANS now puts us in the position to correlate the evolution of mesoscale domain wall patterning with bulk magnetic response functions, which is the overarching goal of the current work. To this end, we performed a series of magnetization and magnetocapacitance measurements as a function of temperature, field, and time. These response functions were chosen due to the potential for future application and to reflect reports of anomalous behaviors in the literature. We chose to track these quantities as a function of time in order to capture the effects of domain wall motion, which we will show has a characteristic timescale on the order of several minutes. We performed measurements on the same samples as used for neutron studies, a crucial step to avoid potential sample dependence in these nonequilibrium processes.

**Figure 5:**
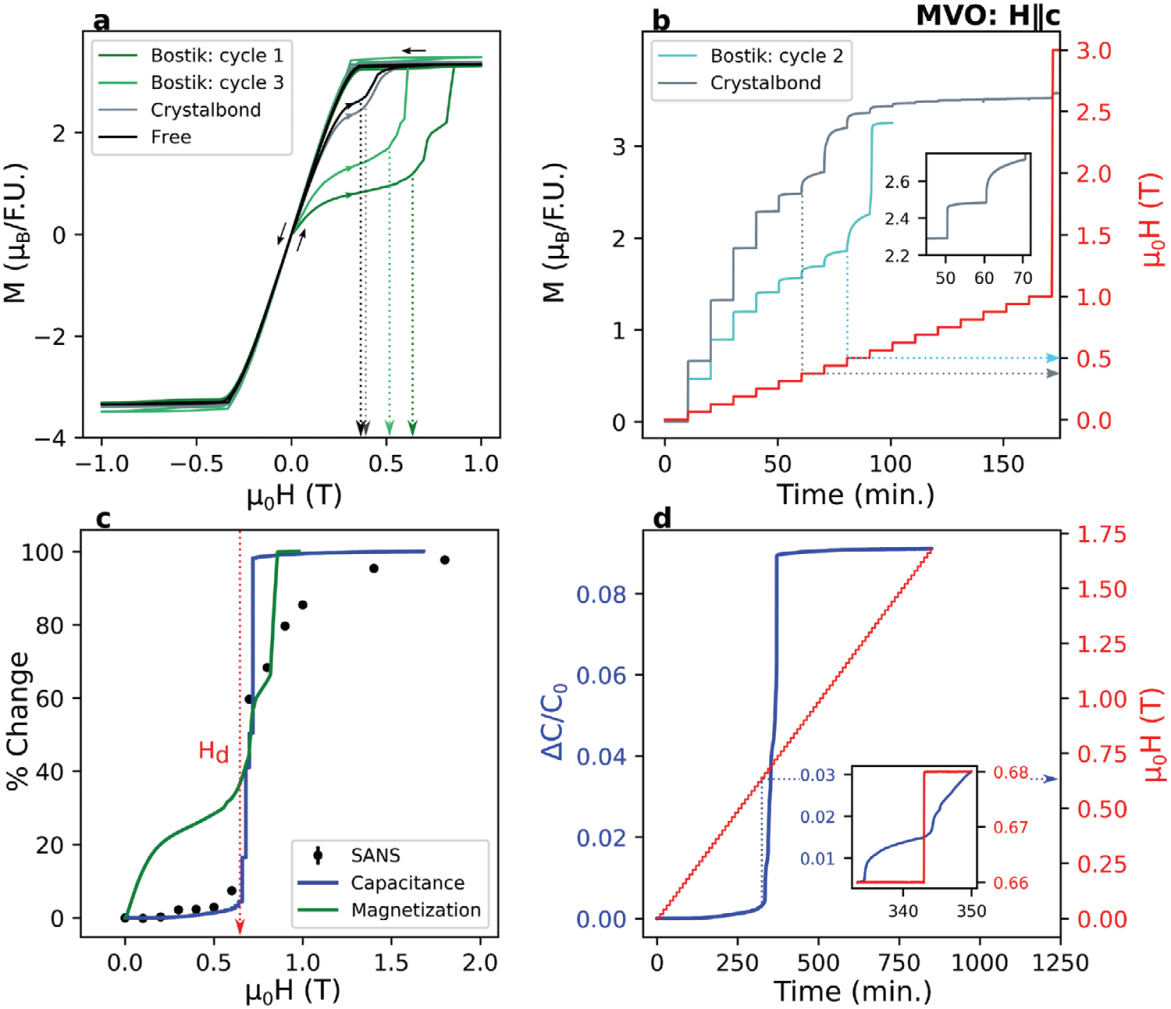
Connection between stripe domains and bulk response functions. Dashed lines indicate the inferred location of *H*_d_. a) Magnetic hysteresis behavior of MVO at 2 K under various mounting conditions with sample either allowed to freely contract, or glued to the mount with Crystalbond adhesive or Bostik superglue. Two Bostik field‐temperature cycles as described in the main text are shown. b) Time‐dependent magnetization in which field is ramped in 60 mT steps to observe slow relaxations associated with domain wall motion. The inset shows a closer look at co‐existing fast and slow relaxation behavior in the Crystalbond‐mounted sample. c) Magnetic field‐induced change in the quantities measured by our different probes (i.e., SANS integrated fin intensity, virgin magnetization, capacitance), plotted as a percentage of total change versus field. d) Measurement of sample capacitance undergoing time‐dependent field ramps (10 mT steps), plotted as a fractional difference ($\frac{\Delta C}{C_{0}}$) from the zero‐field cooled capacitance value (*C*_0_). The inset shows the slow relaxations we associate with low‐temperature stripe domain wall motion.

A subset of this data for MVO is shown in **Figure** **5**. As discussed below, a detailed comparison to the SANS data from the previous section unambiguously establishes a causal connection between the presence and motion of mesoscale stripe domain walls with anomalous magnetic responses in both spin and charge channels. In Figure 5a,b, we show the magnetization of MVO at 2 K with field H∥c (the global axis normal to the surface of the sample mount). Figure 5a displays applied field hysteresis curves for several different choices in how to mount the crystal. For three of the curves, the crystal is glued to the sample mount with different commercially available adhesives, described below. For the last curve, the crystal was held in place by Teflon tape and free to expand or contract without experiencing stress from the mount. In all mounting configurations, zero‐field cooled initial (“virgin”) magnetization curves behave in the same general way: they show an initial sharp increase followed by an extended plateau region. This plateau region terminates at a field which we associate with the stripe wall depinning field *H*_d_, first encountered in Section 2.2 . Above *H*_d_ there is a more rapid positive change in response until saturation. The main effects of varying the mounting strategy are to change the magnitude of the depinning field *H*_d_ and the size of the hysteresis loop (deviation from saturation). When attached with Bostik superglue (green), the anomaly occurs at *μ*_0_ *H*_d_ ≈0.6 T, which is the same field where fin scattering begins to decrease in SANS measurements under similar mounting conditions. When mounted with the adhesive Crystalbond (gray), *H*_d_ is suppressed and the size of the virgin hysteresis is considerably smaller. The freely mounted sample (black) shows marginal reduction of the hysteresis loop as compared with the Crystalbond configuration. Subsequent cycles of temperature and field systematically reduce the Bostik virgin hysteresis loop, as shown in Figure 5a. This is discussed further in subsequent paragraphs.

Tellingly, the act of driving the field above the depinning field (*H* = *H*_d_) is also associated with a dramatic change in the timescale of the magnetization process. This is seen in Figure 5b, which shows magnetization versus time (gray/cyan) as applied field is ramped up in a series of sharp steps (red). Below *H*_d_, the magnetization approaches steady state at a rate faster than the time resolution of the instrument (<1 s). In this field range, we presume the stripe domain wall patterns to be frozen in place. Above *H*_d_, where stripe domain wall motion is inferred from SANS, the response proceeds with a slower characteristic timescale, typically tens of minutes. Once saturated from the virgin state, further hysteresis loops are typical of a soft ferromagnet, with little coercivity, remnant magnetization, or appearance of slow relaxation timescales. Comparable data for MMO can be found in Section S6, Supporting Information.

To further highlight the correlation between the evolution of mesoscale heterogeneity and the bulk response, we overplot in Figure  5c the change in integrated fin intensity from SANS with the virgin magnetization curve from Figure  5a when the crystal was secured with Bostik. Here, magnetization is plotted as a percentage of the total change. It is notable that the inferred depinning field is comparable for both measurements. If one associates the virgin deviation in magnetization with the pinning of domain walls, then this plot indicates that bulk magnetic properties of our materials are determined primarily by the motion of quasi‐1D stripe domains, rather than the orientation of local spins or micron sized demagnetization domains as is typical for classic ferromagnets.^[^ ^1^ ^]^

Perhaps more surprisingly, a similar correlation is found between the evolution of stripe domains and the dielectric response of the material. The blue curve in Figure 5c represents the magnetocapacitance on the same MVO sample at *T* = 3 K, measured using the conventional parallel‐plate capacitor method with electric and magnetic fields aligned along global c. Much like magnetization, magnetocapacitance exhibits a distinct jump at the same *H* = *H*_d_ inferred from the SANS intensity. Figure 5d displays time‐dependent measurements revealing slow relaxations with a timescale comparable to magnetization for *H* > *H*_d_. Moreover the field dependence of the virgin curve exhibits a 9.1% relative increase in capacitance, 60 times larger than subsequent hysteresis loops where SANS and magnetization signatures of the stripes are also removed. Complementary capacitance data showing in‐phase and out‐of‐phase capacitance signals over the entire hysteresis loop, as well as confirmation of a large sample resistance ( 6 TΩ below 20 K) are reported in Section S9, Supporting Information.

These measurements directly connect the magnetocapacitive effects to magnetization and magnetic stripe domains, but cannot unambiguously disentangle capacitive contributions from magnetoelastic sample contraction and purely magnetodielectric effects. Published macrostrain and diffraction measurements^[^ ^23^, ^32^, ^35^ ^]^ allow us to place an upper bound on changes in the sample dimensions, however. Such changes in dimension are often attributed to the redistribution of tetragonal domains. In the extreme case of a single domain crystal (lattice constants *a* = 8.53 Å and *c* = 8.42 Å)^[^ ^23^ ^]^ with *L*_Tet._ c perpendicular to field switching entirely to a different single‐domain state with *L*_Tet._ c uniformly parallel with field, one would only expect a capacitance increase of 2.6%. From this, we estimate an overall field‐induced dielectric constant change of >6.3% relative to the zero‐field cooled value tied to the domain response, the large majority of the magnetocapacitive response.

The exact mechanism behind these observed magnetodielectric effects is as yet unknown, but one can speculate based on what is known about the material. For example, similar magnetodielectric phenomena can arise in nonpolar insulators from a coupling between optical phonons and local spins.^[^ ^12^ ^]^ At low temperature, MVO^[^ ^32^, ^45^, ^46^ ^]^ develops orbital order on the vanadium sublattice, greatly enhancing the spin–lattice coupling in this material. One might expect that phonons coupling to the resulting antiferromagnetic modulation would produce a highly anisotropic dielectric tensor with respect to the *L*_Tet._ c‐axis; field‐induced rearrangement of the *L*_Tet._ c‐axis domain structure would then yield large changes in the macroscopic capacitance far below the ordering transition.

Alternatively, interfacial capacitances at the domain walls may also contribute, arising either from a spatially inhomogeneous dielectric tensor^[^ ^11^, ^17^ ^]^ or domain wall polarity due to bulk structural distortions.^[^ ^47^ ^]^ Our large measured sample resistance likely invalidates models invoking magnetoconduction,^[^ ^17^ ^]^ though this method cannot rule out an insulating surface layer.

### 2.4 The Effect of Strain

Local strain appears to be the key condition for the formation of mesoscale stripe patterning of domains in MVO and MMO. This was first noted in our previous publication,^[^ ^25^ ^]^ but backed by our observation in Section 2.1 that stripe domain walls are orientated in a way consistent with structural twinning. Consistent with the conclusions of this paper, the potential role of applied stress has also been mentioned in many reports of anomalous response functions in the literature.^[^ ^23^, ^35^, ^46^ ^]^ This is also borne out by our own bulk response data presented in Section 2.3. The magnetization data presented in Figure 5a,b, for example, show that “glued” virgin curves display suppressed initial susceptibilities and higher saturation fields relative to the sample which was allowed to expand or contract free from contact with the mount. The strain in MVO crystals is presumed here to be a result of differential thermal contraction between the sample and the sample mount, along with the elastic properties and binding strength of the particular adhesive being used. In this scenario, field and temperature cycling our sample without remounting would be expected to relieve strain, as microcracks form in the adhesive layers. Consistently, we observe a systematic reduction of the size of the magnetic hysteresis loop and *H*_d_ in Figure 5a as temperature and field are cycled. Our SANS data (Figure 4d) similarly revealed a notable decrease in both *H*_d_ and integrated intensities of fin scattering between the first and third field/temperature cycles for MVO.

**Figure 6:**
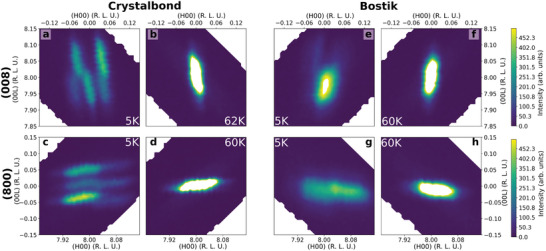
Domain‐related peak splitting induced by mounting stress below and above the ordering transitions. Panels show 2D reciprocal space intensity maps of each peak, integrated from ‐0.25 to 0.25 reciprocal lattice units (R. L. U.) along the (0K0) direction. a,b) (008) peak for Crystalbond mounting. c,d) (800) peak for Crystalbond mounting. e,f) (008) peak for Bostik mounting. g,h) (800) peak for Bostik mounting.

To follow up on these ideas and directly confirm the significant role of mounting configuration on the domain distribution in MVO, we additionally performed a series of conventional diffraction measurements using the wide‐angle neutron diffractomer (WAND^2^) at the high flux isotope reactor (HFIR). In such an experiment, one can often observe the splitting of specific Bragg peaks below a symmetry lowering structural transition into multiple smaller peaks, each associated with a unique domain. Average strain can be inferred from the relative intensities and positions of these smaller peaks. The main results of our measurements on WAND^2^ are given in **Figure** **6**, which shows 2D reciprocal space maps of the (008) and (800) reflections for the same sample as previous measurements. For ease of association, we index reciprocal space locations using the high‐symmetry cubic unit cell at 60 K, with the global (00L) direction defined as normal to the mounting plate, similarly to prior measurements. Each reflection shown here is from a crystal attached to aluminum blades using the Crystalbond (a–d) and Bostik (e–h) polymer adhesives discussed above.

In each case, high‐temperature cubic peaks (Figure 6b,d,f,h) split into several satellite reflections as the crystal cools below $T_{\text{FM}}^{\text{MVO}}$ and develops transformation twins.^[^ ^2^, ^23^ ^]^ Here, the various domains can be identified by satellites which correspond to values of the local tetragonal lattice parameters *L*_Tet._ *a* or *L*_Tet._ *c*, respectively, below or above the parent cubic peak in *Q*.

Data from the Crystalbond mount, inferred from magnetometry to represent close‐to‐ambient conditions, sport noncollinear satellite reflections at the (008) (Figure 6a) and (800) (Figure 6c) locations, corresponding to misaligned *L*_Tet._ **c**‐axis domains. The noncollinearity of the reflections is a form of shear strain common to transformation twins, as neighboring domains tilt to minimize strain energies from the structural transition.^[^ ^2^ ^]^ In contrast, the Bostik‐mounted reflections are broadened below the phase transition, but satellites remain largely collinear with the original principal axes. The out‐of‐plane lattice parameter at the (008) peak (Figure 6e) centers mostly around the value for the transformed *L*_Tet._ *a*, with only weak *L*_Tet._ *c* satellites. The in‐plane (800) (Figure 6g) position shows a single, broad peak with periodicities spanning from *L*_Tet._ *a* to *L*_Tet._ *c*. These data are indicative of strong mounting stresses forcing the microscopic *L*_Tet._ **c**‐axes to lie largely in‐plane. Meanwhile, the collinearity of the resulting peaks is a result of this constraint on domain tilting. This also demonstrates that a net compressive strain is effectively induced by the Bostik in‐plane, as compared with the Crystalbond‐mounted sample. This high‐strain regime of coplanar *L*_Tet._ **c**‐axis domains appears under the same conditions as our observed mesoscopic stripe behavior and the anomalous bulk behaviors in MVO.

With these insights, some of our bulk measurements are more readily understood. Switching from Crystalbond to Bostik, which reduces the crystal volume fraction with the local tetragonal c‐axis parallel to (00L), also increases the volume fraction with the *L*_Tet._ c‐axis perpendicular to applied field in our measurements. In our magnetization measurements, the size of the moment change immediately after *H*_d_ increases at the expense of the moment change which occurs before *H*_d_. Likewise, in time‐dependent measurements the total magnetization change occurring on the fast timescale decreases in favor of the slow‐timescale relaxations. This is all consistent with the quickly relaxing component below *H*_d_ corresponding to the domains with the *L*_Tet._ *c*||(00*L*)||*H*, and the slowly relaxing component, which we associate with the stripes, corresponding to domains with *L*_Tet._ *c*⊥*H*.

This picture is not unrelated to the traditional magnetostrictive effects that can occur in any ferromagnet. Conventionally, the magnetocrystalline anisotropy energy induces local strain wherever magnetic moments deviate from the local easy axes due to internal or applied fields.^[^ ^1^, ^48^ ^]^ As significant deformations of the crystal structure come at large energy costs, conventional magnetostriction effects are typically small (Δ*L*/*L* ≈ 10^−6^) in comparison to the induced strains reported in MMO and MVO (Δ*L*/*L* ≈ 10^−3^).^[^ ^23^, ^24^, ^35^ ^]^ In our presumed explanation, the field‐induced rearrangement of *L*_Tet._ *c* yields an alternate route towards minimizing these same magnetocrystalline and Zeeman energies and yields enhanced effects. Regardless, the large strain and magnetization gradients involved in such a nanoscale magnetostructural modulations mean that internal magnetocrystalline energies may play a significant role in the patterning behavior.

The observed increase in *H*_d_ and the robustness of the stripes against field is more difficult to understand. One effect that may play a role is any alteration of the demagnetizing field (dipolar interaction) caused by a suppression of domains that start in the *L*_Tet._ *c*||(00*L*)||*H* configuration. Another likely possibility is an intrinsic change in the ferroelastic activation energy^[^ ^41^ ^]^ due to increased strain or magnetocrystalline energies at the domain walls in moving from a 3D to a 2D domain structure. We believe this is not unlikely, especially given the qualitative change from misaligned to collinear peak splitting as described above.

Further systematic studies would be required to get deeper insight into these phenomena. Such studies may include a comprehensive mapping of this domain wall behavior as a function of applied stress. Our coarse control of applied stress, while not dissimilar to the use of epitaxial strain to engineer thin films,^[^ ^49^, ^50^ ^]^ leaves room for more quantitative future strain‐cell experiments.

In MMO, the situation is more subtle but likely closely related. For this material, neither SANS patterns nor bulk response functions are strongly dependent on mounting configuration, but vary strongly with the method employed to grow single crystals. Due to the presence of a Jahn–Teller transition at *T*_JT_=1440 K, crystals grown from the liquid phase using the optical floating‐zone techniques pass through a dramatic symmetry lowering structural phase transition when cooling from growth conditions. Consequently, these crystals inherit an intrinsically strained tetragonal twin structure at room temperature.^[^ ^40^ ^]^ Further changes in structure and ordering of spins occur within these tetragonal twins. MFM surface maps associate high strain regions in MMO with shorter‐wavelength stripe modulations.^[^ ^25^ ^]^ The application of further mounting stress is then not needed to stabilize either stripe domains or see unusual response behaviors.

Confirming this idea, low‐strain crystals obtained by us from flux growths at temperatures below *T*_JT_ demonstrate markedly different bulk properties.^[^ ^40^ ^]^ At the same time, SANS patterns show a dramatic suppression of fin features associated with mesoscale stripe patterning.

In the highly strained MMO crystal, we see many of the signatures of domain wall depinning with applied field as in MVO. This includes a cross‐over from fast to slow magnetization timescales in the virgin magnetization curves at the same field region when we see a decrease in SANS intensity. In contrast with MVO however, the complete suppression of scattering intensity does not seem to be associated with a single depinning field *H*_d_, but rather a distribution of depinning fields. As *H*_d_ appears highly responsive to stress perturbations, this may be related to the broad distribution of strain fields induced during crystal growth. Additionally, the stripe patterns in MMO may have lower activation energies due to the relatively small mismatch between relevant orthorhombic lattice parameters (*L*_Orth._ $\frac{a}{b}\approx 99.4\%$),^[^ ^22^ ^]^ as compared to MVO's larger mismatch between tetragonal directions (*L*_Tet._ $\frac{a}{c}\approx 98.7\%$). Bulk magnetic measurements and neutron characterizations on the flux grown crystals are reported in Sections S6 and S8, Supporting Information.

## 3 Conclusion

The collective data presented above paint a clear and unambiguous picture in which formation and movement of magnetization domain walls in the bulk dominates both the low‐temperature spin and charge response functions in two different ferrimagnetic spinels. These include not only conventional ferromagnetic domains driven by dipole fields but, crucially, anomalous stripe‐like domains which form below known magnetostructural transitions in both materials. Our SANS investigations of crystals previously explored with surface probes such as MFM show that these stripe domains extend to the bulk, and further reveal that they respond to applied magnetic fields above a highly anisotropic critical depinning field, *H*_d_.

Atypical of ferromagnets, the lengthscale and directionality of these domains bear unmistakable similarity to the structural domains observed in high‐temperature ferroelectric materials. This suggests the stripes contain a structural component, with patterns forming to reduce long‐range strain fields. The small elastic perturbations necessary to alter the domain structure and response functions point to stress as a useful control knob of magnetic properties in these materials. The role of lattice degrees of freedom in these domains provide a natural interpretation for both the emergent slow timescales below the magnetostructural transitions and the magnetocapacitive effects reported above.

The unique characteristics of ferrimagnetic materials make them ideal candidates to demonstrate anomalous behaviour of this kind. Antiferromagnetic spin correlations couple strongly to optical phonons to enhance spin–lattice coupling,^[^ ^12^ ^]^ while the net ordered moment allows for coupling to external magnetic fields.

Similar phenomenologies are expected for other materials with magnetostructural transitions, extending to other novel degrees of freedom and higher transition temperatures. One example is similar behavior observed below the Verwey transition (*T* = 125 K) in magnetite,^[^ ^27^ ^]^ an inverse spinel multiferroic with unique local physics. Other ferrimagnetic spinels also display a broad selection of novel electronic properties^[^ ^51^, ^52^ ^]^ and pressure‐induced electron itinerancy^[^ ^53^, ^54^ ^]^ which could couple to such domain effects. Our observations open up exciting new possibilities for tunable functionality in materials with spin–lattice coupling through manipulation of magnetostructural domains.

## 4 Experimental Section

### Sample Preparation

Single‐crystal samples of both materials were grown using the traveling‐solvent floating zone method, as described in the previous work.^[^ ^25^, ^45^ ^]^ The samples were cut into a plate‐like geometry with thickness ranging from 0.1 to 1 mm to reduce multiple scattering and absorption effects in SANS measurements. For MMO, additional crystals were grown at temperatures below the known cubic–tetragonal Jahn–Teller transition using anhydrous sodium tetraborate (Borax) flux, in order to reduce tetragonal domain formation and thus internal strain. SANS results on this low‐strain crystal showed a substantial reduction in fin scattering, as shown in Section S9, Supporting Information . Additional details regarding the two growth methods and the resulting strain effects for this material are given elsewhere.^[^ ^40^ ^]^

The neutron scattering and magnetization measurements exhibited clear differences in the MVO domain structure depending on the strain induced by the choice of mounting method. Strained measurements were obtained by using commercially available adhesives Crystalbond and Bostik superglue to attach samples to an aluminum backing plate. Low‐strain magnetization measurements were obtained by suspending the sample using teflon tape (held in place by the sample's plate‐like geometry), enabling free thermal contraction.

### Magnetic Force Microscopy

MFM imaging was performed at the University of Illinois Materials Research Laboratory as detailed in our previous paper.^[^ ^25^ ^]^ Imaging of surfaces normal to c for each crystal at low temperature and under magnetic field was directly comparable to the SANS measurements. Samples were glued to copper mounts using Stycast 2850FT Epoxy, which created a finite sample strain comparable to those in the SANS measurements.

### Small‐Angle Neutron Scattering

The neutron scattering work was carried out at GP‐SANS in the HFIR at Oak Ridge National Laboratory.^[^ ^55^ ^]^ The bulk of the work was done with the neutron beam parallel to the global c‐axis (normal to the sample plate) to probe each material in the (HK0) scattering plane to capture the known stripe propagation direction. A series of discrete wavelengths between 4.76 Å and 19 Å with a $\frac{\Delta \lambda }{\lambda }$ of 0.13 was used to access *Q* between 0.001 and 0.1 Å^−1^. As described in Section S3, Supporting Information, finely spaced rocking scans were obtained where necessary to account for the highly anisotropic nature of fin scattering in the out‐of‐plane direction of the detector.^[^ ^37^ ^]^ A horizontal field magnet was used to place magnetic field along the incident neutron beam, with temperature control achieved either by a variable temperature insert (base *T* = 1.5 K) or a Helium‐4 Closed‐Cycle Refrigerator (base *T* = 5 K). All SANS data presented in this manuscript corresponded to differences between low‐temperature measurements and high‐temperature backgrounds taken in the paramagnetic phase of each material. The resulting 2D datasets were analyzed using 1D cuts. Integrated intensities tracking the different scattering components were obtained by fitting the annular Q‐cuts to Lorentzian profiles. Likewise, *I* versus *Q* along specific fin directions were obtained using the rectangular cuts displayed in Figure S2, Supporting Information, after the geometric correction detailed in Section S3, Supporting Information was applied to rocking curves. In MMO, these geometric corrections were complicated by the mosaicity inherent in the sample, and the intensity distributions represent cuts of 2D data taken with one orientation.

### Magnetometry

SQUID magnetometry was carried out inside a Quantum Design Magnetic Properties Measurement System (QD MPMS 3) or an MPMS XL as for MMO in Section S5, Supporting Information. DC measurements were performed for field and temperature sweeps using the standard DC scan mode. For time‐dependent measurements, the vibrating sample magnetometer (VSM) option of the MPMS 3 was used, in order to obtain the highest possible time resolution. To observe the time‐dependent relaxations, field steps employed the highest available ramp rate (700 Oe s^−1^).

### Magnetocapacitance Measurements

Magnetocapacitance measurements were performed inside a Quantum Design Physical Properties Measurement System with a home‐built insert at the National High Magnetic Field Laboratory at Los Alamos National Laboratory. To probe the magnetocapacitance effect, the capacitance of the MVO sample was measured with an Andeen‐Hagerling AH‐2500A commercial capacitance bridge at a driving frequency of 1000 Hz and amplitude of 15 V. Silver paint and gold wire were used as contacts on the parallel faces of the plate‐like (1.5 mm × 3 mm × 0.1 mm) MVO sample. A calibrated Cernox sensor near the sample was used to monitor temperature. The resistance was measured using a Keithley 6517A electrometer with a bias voltage of 10 V, with the same mounting configuration and probe as the capacitance measurements.

### Neutron Diffraction

Neutron diffraction was performed at the wide‐angle neutron diffractometer (HB‐2C, WAND^2^) at HFIR. The experiment was performed using a wavelength of 1.5 Å, again using a Helium‐4 CCR for temperature control. The two different mounting configurations were achieved by gluing the sample to an aluminum plate using either Crystalbond or Bostik adhesives, with the crystal aligned such that both in‐plane and out‐of‐plane directions could be probed for comparison.

### Statistical Analysis

1) Pre‐processing of data: The statistics of neutron data were determined by Poisson counting statistics, which depend on the incoming neutron flux, the strength of scattering, and the integration time for each data point. As such, the integration time of neutron measurements was fixed to collect sufficient statistics on each feature of interest. The raw intensities and error bars were normalized by the standard correction factors for SANS: this normalization factor included detector efficiency, a geometric solid‐angle factor to account for angular acceptance of each detector tube, and the incoming neutron flux from the reactor. 2) Data presentation: An error bar of one standard deviation (as determined by Poisson statistics) was shown wherever applicable, as described in figure captions where relevant. All fit information as given in the text and figures also included standard deviation error bars as determined by nonlinear least‐squares fitting. 3) Sample size: SANS results were reproduced on four samples of MMO and two samples of MVO, each cut from the same original boule. Individual curves were the product of measurement on a single sample. 4) Statistical methods: Neutron scattering method was inherently a statistical probe which integrated over the full sample. All statistical (Poisson) uncertainties inherent to the data were propagated through each processing step. For fits, statistical error‐bars were obtained from the obtained covariance matrix, and accounted for any correlations between parameters. 5) Data reduction (normalization) was performed using the drtSANS package developed at Oak Ridge National Laboratory. The Python libraries pandas^[^ ^56^, ^57^ ^]^ and lmfit^[^ ^58^ ^]^ were used for further analysis and curve fitting, and matplotlib^[^ ^59^ ^]^ for data visualization.

## Conflict of Interest

The authors declare no conflict of interest.

## Supporting information

Supporting Information

## Data Availability

The data that support the findings of this study are available from the corresponding author upon reasonable request.
