# Domain Wall Patterning and Giant Response Functions in Ferrimagnetic Spinels

**DOI:** 10.1002/advs.202200207

**Published:** 2022-02-24

**Authors:** Lazar L. Kish, Alex Thaler, Minseong Lee, Alexander V. Zakrzewski, Dalmau Reig‐i‐Plessis, Brian A. Wolin, Xu Wang, Kenneth C. Littrell, Raffi Budakian, Haidong Zhou, Zheng Gai, Matthias D. Frontzek, Vivien S. Zapf, Adam A. Aczel, Lisa DeBeer‐Schmitt, Gregory J. MacDougall

*Adv. Sci*. **2021**, *8*, 2101402

DOI: 10.1002/advs.202101402

In the originally published version of article, the affiliations of Haidong Zhou are incorrect. Please find the correct affiliations here:

L. L. Kish, A. Thaler, A. V. Zakrzewski, D. Reig‐i‐Plessis, B. A. Wolin,

X. Wang, R. Budakian, G. J. MacDougall

Department of Physics and Materials Research Laboratory

University of Illinois at Urbana‐Champaign

Urbana, IL 61801, USA

E‐mail: lazark2@illinois.edu; gmacdoug@illinois.edu

M. Lee, V. S. Zapf

National High Magnetic Field Laboratory

Los Alamos National Laboratory

Los Alamos, NM 87544, USA

D. Reig‐i‐Plessis

Department of Physics and Astronomy and Quantum Matter Institute

University of British Columbia

Vancouver, British Columbia V6T 1Z1, Canada

H. Zhou

Department of Physics and Astronomy University of Tennessee

Knoxville, Tennessee 37996, USA

R. Budakian

Department of Physics and Astronomy

University of Waterloo

Waterloo, Ontario N2L 3G1, Canada

Z. Gai

Center for Nanophase Materials Sciences

Oak Ridge National Laboratory

Oak Ridge, TN 37831, USA

A. Thaler, K. C. Littrell, M. D. Frontzek, A. A. Aczel, L. DeBeer‐Schmitt

Neutron Scattering Division

Oak Ridge National Laboratory

Oak Ridge, TN 37831, USA

E‐mail: debeerschmlm@ornl.gov

