# Supplementary material for: Domain Wall Patterning and Giant Response Functions in Ferrimagnetic Spinels
Source: Adv Sci (Weinh). 2021 Nov 1;8(23):2101402. doi: 10.1002/advs.202101402 (PMC8655211; doi:10.1002/advs.202101402)
Supplement: Supplementary file 1 — Supporting Information [file ADVS-8-2101402-s001.pdf]

Supporting Information: Domain wall patterning and giant response functions in ferrimagnetic spinels

L. L. Kish,[1,*] A. Thaler,[1,2] M. Lee,[3] A. V. Zakrzewski,[1] D. Reig-i-Plessis,[4] B. Wolin,[1] X. Wang,[1] K.C. Littrell,[2] R. Budakian,[1,5] H. D. Zhou,[6] Z. Gai,[7] M. D. Frontzek,[2] V. S. Zapf,[3] A. A. Aczel,[2] L. DeBeer-Schmitt,[2] and G. J. MacDougall[1]

[1]*Department of Physics and Materials Research Laboratory, University of Illinois at Urbana-Champaign, Urbana, Illinois 61801, USA*

[2]*Neutron Scattering Division, Oak Ridge National Laboratory, Oak Ridge, Tennessee 37831, USA*

[3]*National High Magnetic Field Laboratory, Los Alamos National Laboratory, Los Alamos, New Mexico 87544, USA*

[4]*Department of Physics and Astronomy and Quantum Matter Institute, University of British Columbia, Vancouver, BC V6T 1Z1, Canada*

[5]*Department of Physics and Astronomy, University of Waterloo, Waterloo, Ontario N2L 3G1, Canada*

[6]*Department of Physics and Astronomy, University of Tennessee, Knoxville, Tennessee 37996, USA*

[7]*Center for Nanophase Materials Sciences, Oak Ridge, Tennessee 37831, USA*

(Dated: July 29, 2021)

S-1. OVERVIEW OF CRYSTALLOGRAPHIC CONVENTIONS

Here we present an overview of the crystallographic conventions used in this paper. As stated in the text, the two materials do not share space groups at relevant temperatures. Mn_3O_4 (MMO) has the tetragonal spacegroup $I4_1/amd$ at room temperature, and retains this symmetry until 33K whereupon it transitions to one of two orthorhombic phases. MnV_2O_4 (MVO) retains the cubic $Fd\bar{3}m$ spacegroup until 53K, and then transitions to its low-temperature tetragonal $I4_1/a$ symmetry. We wish to highlight the differing conventions for the cubic and tetragonal unit cells. By definition, the tetragonal cell selects a unique axis $\mathbf{c_T}$ along one of three symmetry-equivalent cubic directions $\mathbf{c_C}$. The remaining tetragonal directions $\mathbf{a_T}$ and $\mathbf{b_T}$ are conventionally defined with a 45° rotation away from the cubic directions $\mathbf{a_C}$ and $\mathbf{b_C}$. This is demonstrated in Fig. S-1.

Unless otherwise specified, the text refers to the **a**, **b**, and **c** axes using the high symmetry cubic convention for spacegroup Fd-3m. In part, we do this to account for the fact that the macroscopic domain structures do not globally follow the symmetries of the local unit cell. On cooling in ambient conditions through a symmetry-breaking transition, a developing domain distribution may, on average, retain the parent symmetry of the high-temperature structure. However, since growth and environmental conditions can break this symmetry our chosen **a**, **b**, and **c** directions are not arbitrary. Our floating-zone-grown samples of MMO grow with a uniquely defined tetragonal $\mathbf{c_T}$ axis, chosen to be the **c**-axis in the text. For MVO, in-plane sample strain and magnetic field both serve to define a unique axis normal to the sample plane. We therefore choose our reference **c** to be parallel to the cubic $\mathbf{c_C}$ direction normal to the crystal plate's surface and our applied magnetic field, with the remaining two directions **a** and **b** coplanar with the sample mount. For both materials, the reciprocal lattice vectors are parallel to the real-space directions, as shown in Fig. S-1. The main text respectively denotes the real and reciprocal space directions as "**a**,**b**,**c**" and "$\mathbf{a}^*$,$\mathbf{b}^*$,$\mathbf{c}^*$".

S-2. RECTANGULAR LINE CUTS FOR I VS Q CURVES

To fit a subset of our 2D anisotropic datasets to 1D models, we took rectangular linecuts of the fin scattering parallel to the propagation direction of the feature. The regions of inte-

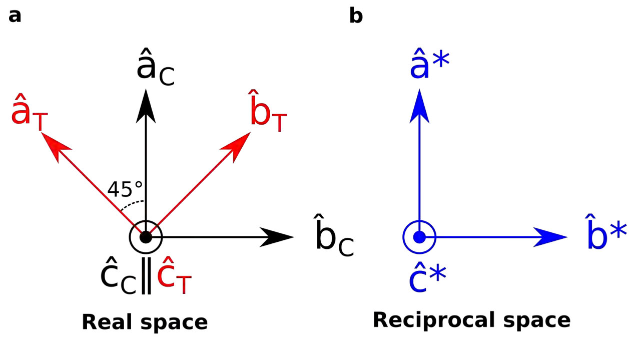

FIG. S-1: Schematic showing our conventions for labeling the different crystal directions. (**a**) Relationship between conventions for cubic and tetragonal unit cells. (**b**) Reciprocal space directions in the tetragonal convention.

gration for these linecuts, with the transverse integration ranges for each instrument setting, are displayed by the blue rectangles in Fig. S-2. The integration region was widened for instrument settings at progressively higher Q, in order to account for resolution broadening of the features.

S-3. GEOMETRIC CORRECTION FOR ANISOTROPIC SCATTERING

Single crystal samples have the potential to display sharply anisotropic scattering, where the geometric effect of Ewald sphere curvature is significant compared to the width of the feature. This must be accounted for in order to accurately fit these data without artificial drop-offs of the intensity with increasing Q. In the case of MMO, this issue is complicated by small misalignments in the grain structure, which result in diverging bands of intensity in the mid-Q and high-Q range.

For MVO, where our crystals are truly single-grain, we are able to fully correct our data for this effect. This was achieved using finely-spaced scans as a function of sample rotation with respect to the incident beam (around the x-axis), to capture the 3-D nature of the scattering. The results are then projected onto the crystal axes in reciprocal space for linecuts. The raw data for MVO, as collected by rectangular cuts in the yz-plane ($\hat{\mathbf{z}}$ normal to detector plane) and the resulting corrected projection are shown in Fig. S-3, with the detector plane represented by the dashed green lines.

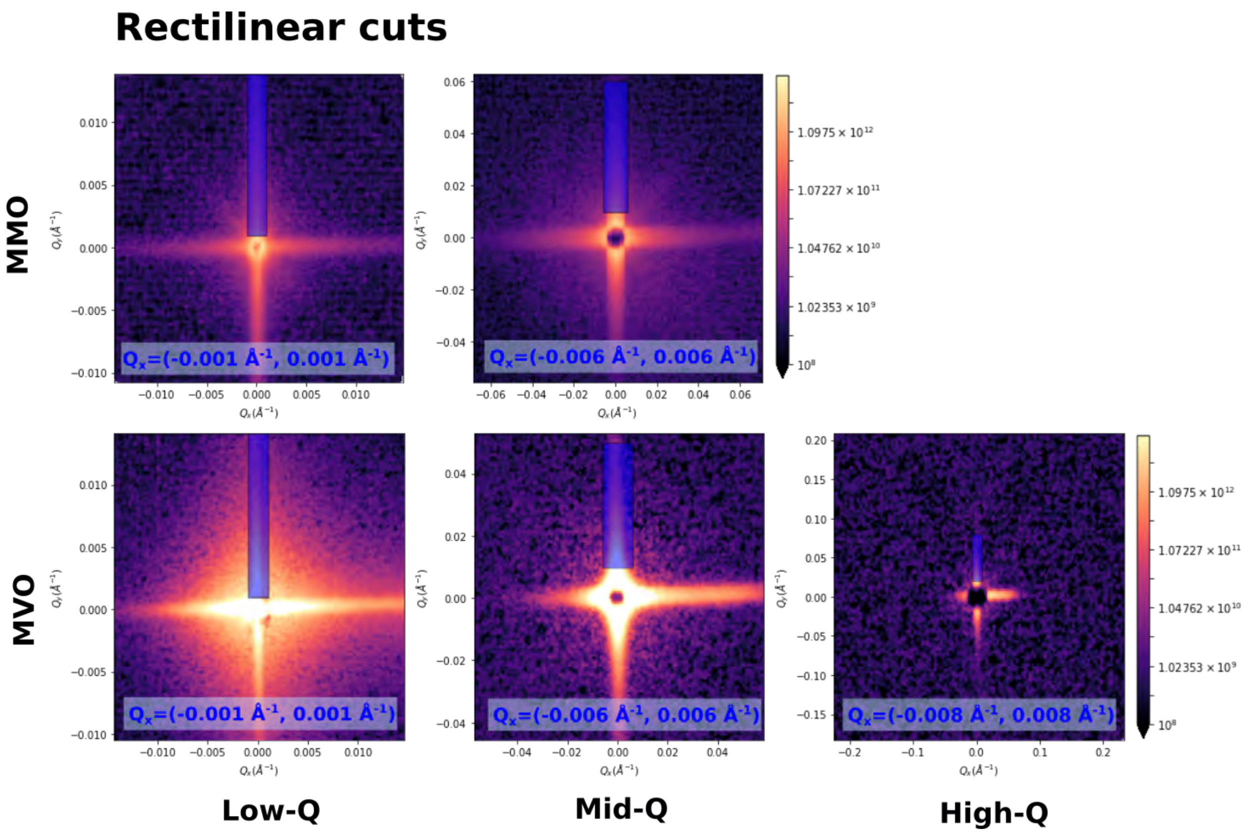

FIG. S-2: **Representations of SANS rectangular cuts.** Rectangular cuts for (**a**) MMO and (**b**) MVO, for each range of Q measured. The horizontal bounds of the integration regions are listed at the bottom of each panel.

S-4. MODELLING OF SANS INTENSITY VS Q

Due to the relative magnitude of the magnetic moment in these samples compared with the expected atomic density fluctuations arising from structural distortions, we assume that nearly all of the SANS intensity is magnetic in origin.

For unpolarized SANS, the cross-section for magnetic scattering is proportional to the squared modulus of the Fourier-transform of the magnetization component perpendicular to $\hat{\mathbf{Q}}$:

$$I_M(\mathbf{Q}) \propto \left| \int d\mathbf{r} \mathbf{M}_{\perp \mathbf{Q}}(\mathbf{r}) e^{i\mathbf{Q}\cdot\mathbf{r}} \right|^2 \tag{1}$$

These measurements cannot distinguish between two distinct microscopic pictures for the stripes. The first, which would correspond to order-disorder magnetic phase coexistence, involves a largely unidirectional magnetization with a spatially varying amplitude. The

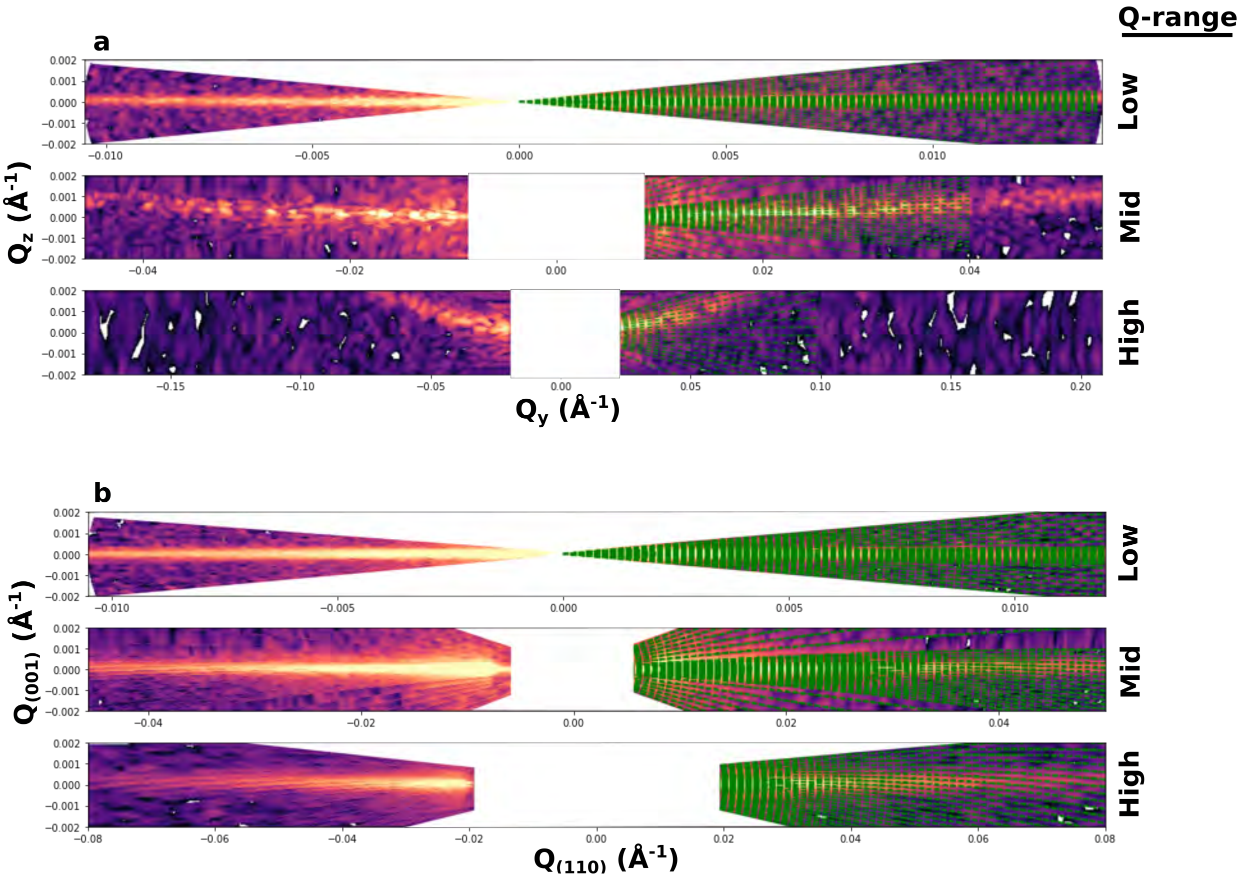

FIG. S-3: **Effects of Ewald sphere curvature and correction for MVO rectangular data.** The 2D panels represent intensity captured by the rectangular linecuts as a function of sample rotation in (**a**) detector coordinates and (**b**) projected onto reciprocal space crystal axes. The green dashed lines represent the detector curvature.

second picture, which corresponds more naturally to nanostructural twin domains, involves a spatially alternating magnetization direction from stripe to stripe as the magnetic easy axis rotates into the perpendicular direction for each variant of the c-axis structural distortion.

Bearing in mind this ambiguity, we assume the former picture for fitting purposes, where the real-space variation of M is constant within the bulk of a domain, and changes linearly within the interfaces. This is represented schematically in Fig. S-4, where the width of a stripe is s_d and the width of its walls are s_w. By the convolution theorem, the Fourier-transform of the magnetization amplitude is simply the product of two sinc squared functions, and scattering from a stripe of this form can be written as:

$$I(Q_{(110)}) = a\ sinc^2\left(\frac{s_d Q_{(110)}}{2}\right) sinc^2\left(\frac{s_w Q_{(110)}}{2}\right). \tag{2}$$

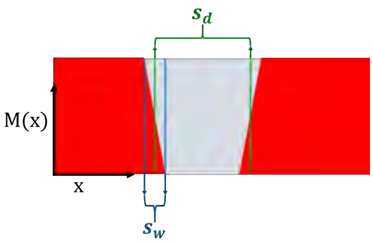

FIG. S-4: **Schematic representation of our model of a solitary stripe in real space.** $M(x)$ represents a spatially-varying scalar component of the magnetization, with stripe width s_d and wall width s_w.

Here, a is a proportionality constant. In order to take into account variations in these length-scales, each sinc squared term is numerically convoluted with a Gaussian size distribution

$$G = \frac{\exp(-\frac{(s-s_*)^2}{2\sigma^2})}{\sqrt{2\pi\sigma}}, \tag{3}$$

with a high-Q cut off at s=0:

$$I(Q_{(110)}) = a\left(sinc^2\left(\frac{sQ_{(110)}}{2}\right) * G(s, s_d, \sigma_d)\right)\left(sinc^2\left(\frac{sQ_{(110)}}{2}\right) * G(s, s_w, \sigma_w)\right). \tag{4}$$

For use in fitting intensities, this model is once more convoluted with the Gaussian resolution function of GP-SANS. In principle, inter-stripe correlations for densely packed stripe domains could generate peaks in the data. However, we found that the single stripe form factor described the variation in Q sufficiently well without additional structure factor components for most of our data. While the lack of a structure factor is a likely effect of high polydispersity, we cannot preclude the possibility of additional structure factor components below the low-Q limits of our measurement. The exception to this was the case of MMO with $\mathbf{H}||\mathbf{c}$, as displayed in the main text, as a weak peak appeared in the data with increasing field. Data in this case was modelled by a combination of Lorentzian and Q^{-4} intensity components, as our measured Q-range was insufficient to produce a unified fit involving structure and form factors.

S-5. MAGNETIC TRANSITIONS AND CHARACTERIZATION

Temperature dependent DC (Fig. S-5 a,b) and AC (Fig. S-5 c,d) magnetization curves show the relevant magnetic transitions in each material. The magnetostructural transitions,

which are associated with an increase of the local magnetic anisotropy, coincide as expected with an increased zero field-cooled-field-cooled splitting in the DC magnetization. The AC susceptibilities observe a peak at the transition then a subsequent drop. In MVO the AC response sees a degree of frequency dependence as well, implying a range of relaxation times associated with the orbital-ordering transition. This behavior is not replicated by the higher temperature spin-only ordering transition.

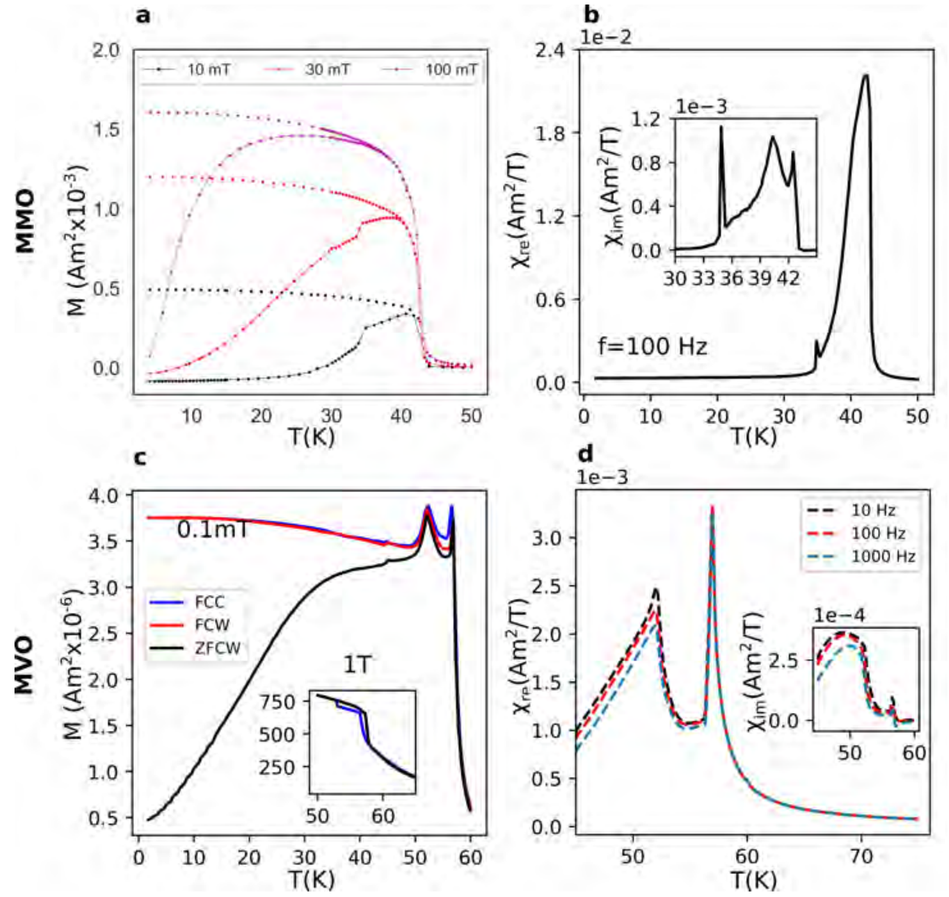

FIG. S-5: **Temperature dependent DC and AC magnetic measurements on MMO and MVO.** Plots show zero-field cooled warming (ZFCW), field-cooled cooling (FCC), and field-cooled warming (FCW) data. **a** MMO DC magnetization at various fields: ZFCW curves are connected lines, FCW curves are dotted symbols. **b** MVO DC susceptibility at low field (inset: high field) **c** MMO AC (ZFCW,H_{AC} = 0.01mT) magnetization, **d** MVO AC (ZFCW, H_{AC} = 0.01mT) susceptibility at various frequencies.

S-6. BULK MAGNETIC RESPONSE OF MMO

In the main text, we associate time-dependent behavior in the virgin hysteresis loops of MVO with motion of magnetostructural domain walls. The prevalence of this region in the hysteresis loop was associated with sample strain. In Fig. S-6 we present analogous magnetization data for MMO, for both high strain floating-zone (F.Z.) and low strain Borax growths.

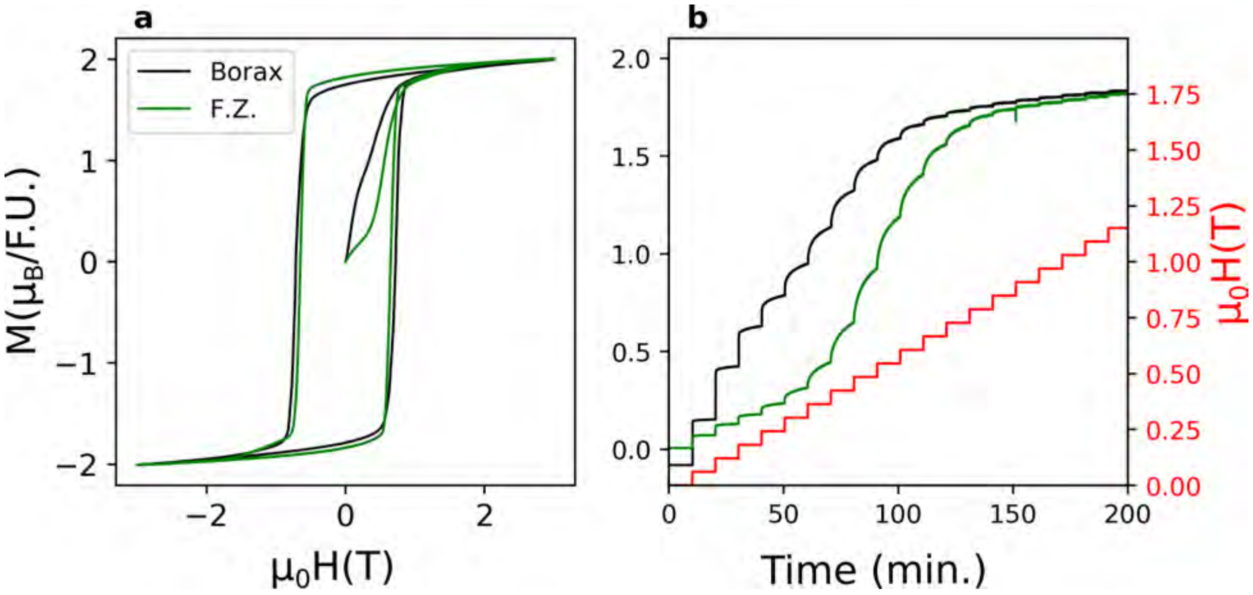

FIG. S-6: **MMO magnetization for two different growth methods.** Magnetization as a function of (**a**) field and (**b**) time for MMO, comparing samples grown using floating-zone and Borax flux methods.

For the sake of comparison, measured Borax moments were rescaled to match the F.Z. extrema. The hysteresis loops are quite similar, with the largest difference once again occurring in the virgin curve. As observed for MVO, a kink in the virgin curve is associated with a crossover in the time dependent magnetization from a fast to a slowly relaxing timescale. It is worth noting that the Borax sample was attached via epoxy to a sample holder for this measurement due to sample geometry. This likely induced mounting stress comparable to the glued case in our MVO measurements.

S-7. SUPPLEMENTAL IN-FIELD SANS IMAGES

This section includes supplemental data for our in-field SANS characterization. Figure S-7 shows fins at low temperature upon application and removal of applied field slightly misaligned from the (001) direction. Fin scattering is completely removed by the highest field (3.5T), and does not return on removal of applied field. Figure S-8 displays 2D patterns taken by rocking curves on increasing the applied magnetic field. Scattering is fully removed by 2.5T, however fields were applied up to 5T. The rightmost panel shows scattering on removal of the applied field. A small amount of very weak anisotropic scattering does return around the beamstop, but it is greatly suppressed compared to the initial pattern.

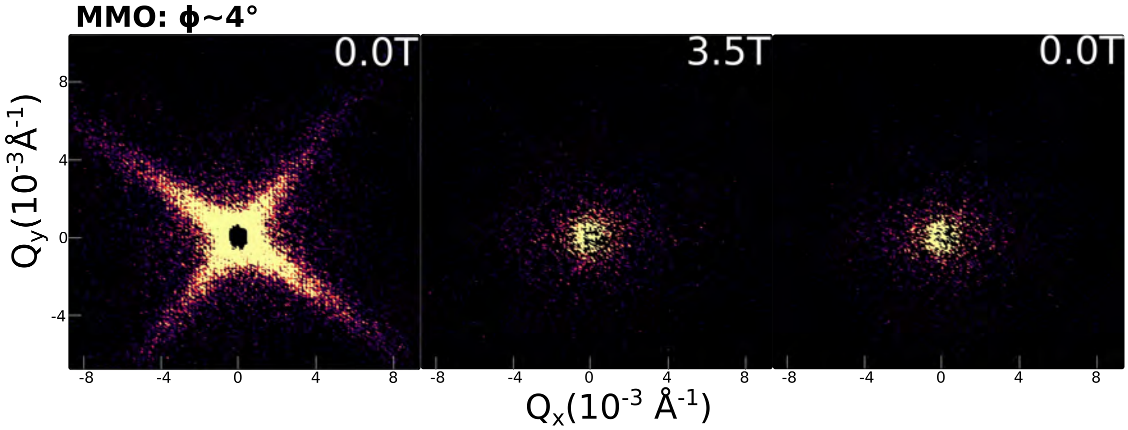

FIG. S-7: **2D SANS images of MMO upon saturation and removal of field.** Images proceed sequentially from left to right. Fin scattering does not return upon removing field once saturated.

S-8. SANS FROM LOW-STRAIN MMO SAMPLE

Our SANS characterization of Borax samples failed to locate any sign of the temperature-dependent fin scattering. Figure S-9 displays a long-count difference pattern between base temperature (T = 1.5K) and background at (T = 49K). The horizontal anisotropic scattering is due to small-angle reflections from the edges of our crystal, and shows none of the characteristic temperature dependence of the stripe domains. Although we cannot say that stripe domains are absent in this crystal, at the very least we demonstrate a dramatic suppression of the effect.

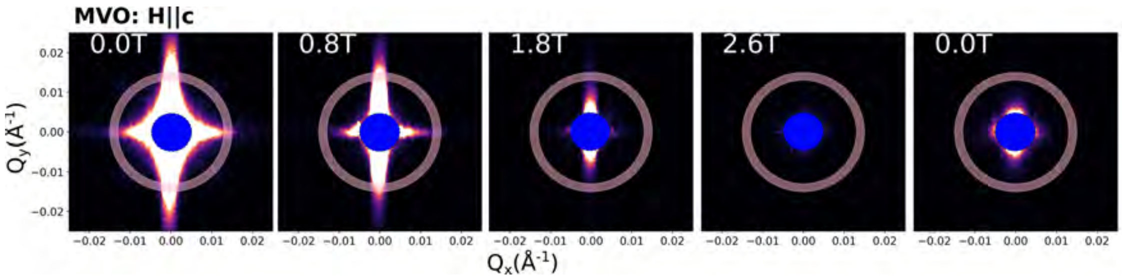

FIG. S-8: **2D SANS images of MVO upon increasing field parallel to the c-axis.** The dark blue circles represent the location of the beamstop which is used to prevent overload of the SANS detector at these wavelength settings. The shaded annular regions represent our annular cuts used to fit data for the order parameter shown in the main text.

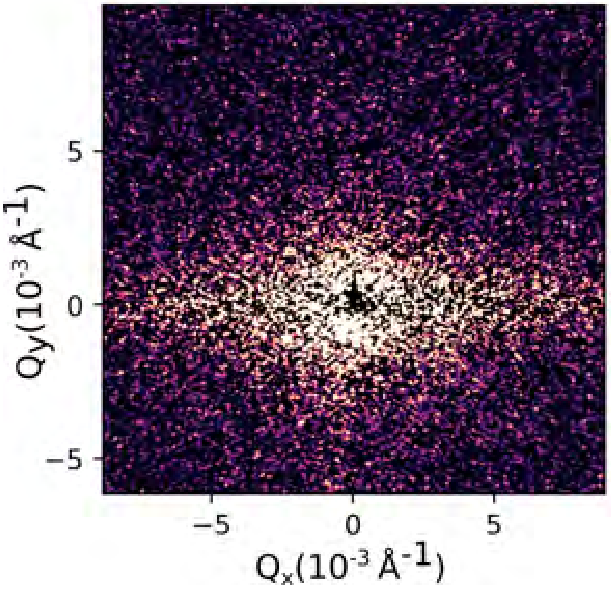

FIG. S-9: **SANS from Borax sample growth.** Measurement was done at T = 1.5K. The plot shows the difference with a high temperature dataset taken in the paramagnetic regime of MMO at T = 49K.

S-9. COMPLEMENTARY CAPAPACITANCE DATA FOR MVO

In this section we present complementary capacitance measurements on MVO at $T = 3$K. Figure S-10a displays the raw in (out of) phase capacitance signals $C'(C'')$ as a function of field, which led to the results shown in the main text. C'' displays a similar jump to C', though both curves likely incorporate the effects of changes in sample geometry. Figure S-

10b shows sample resistance as a function of temperature as referred to from the main text, exceeding 6TΩ at 20K, and increasing beyond our measurement limit at lower temperatures. The low-temperature scatter is insulating background noise. Figure S-10c compares the virgin curve and subsequent capacitive hysteresis loops. The maximum change in the field-cycled loops is a factor of 60 less than that of the virgin curve. Hysteresis loops at various field ramp rates are displayed in Fig. S-10d, showing a small systematic reduction in the size of the response with increased rate. This indicates that MVO retains a small degree of time-dependent behavior in its field-cycled state.

---

* Electronic address: `lazark2@illinois.edu`

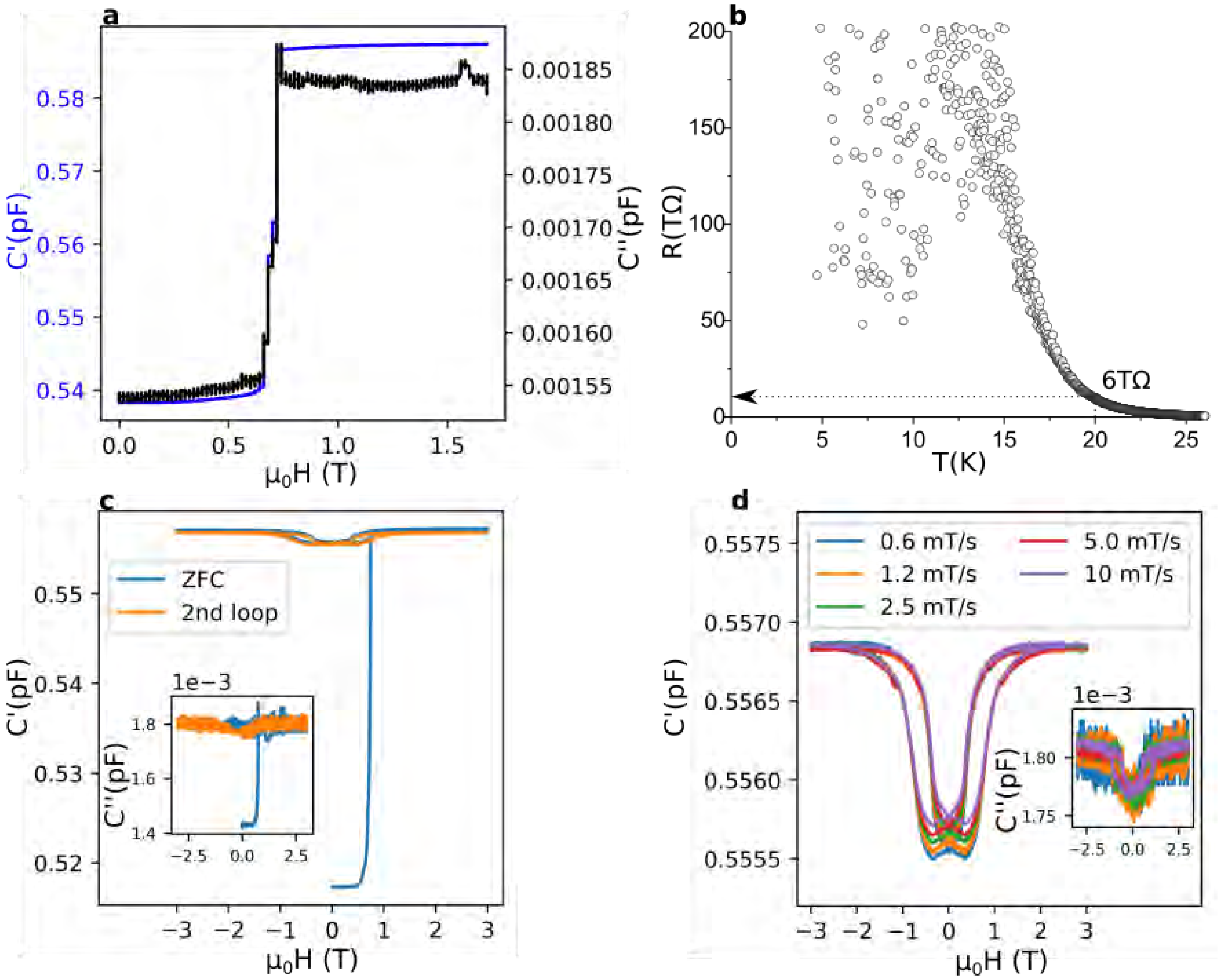

FIG. S-10: **Complementary capacitance data as a function of field and time for MVO.** Measurements were done at $T = 3$K. C' refers to the in-phase signal, while C'' refers to the out-of-phase signal. (**a**) Raw data for the time-dependent measurement in the main text. C" shows a jump of similar to that in C'. (**b**) Temperature dependence of sample resistance. (**c**) Comparison of virgin curve and further hysteresis loops. (**d**) Ramping rate dependence of hysteresis loops, showing systematic reduction of the size of the loop with faster rates.
